# Recent Applications, Challenges, and Future Prospects of Microbial Fuel Cells: A Review

**DOI:** 10.1002/gch2.202500004

**Published:** 2025-04-16

**Authors:** Ripel Chakma, M. Khalid Hossain, Prabhu Paramasivam, R. Bousbih, Mongi Amami, G. F. Ishraque Toki, Rajesh Haldhar, Ashish Kumar Karmaker

**Affiliations:** ^1^ Department of Electrical and Electronic Engineering Dhaka University of Engineering & Technology Gazipur 1707 Bangladesh; ^2^ Institute of Electronics Atomic Energy Research Establishment Bangladesh Atomic Energy Commission Dhaka 1349 Bangladesh; ^3^ Department of Advanced Energy Engineering Science Interdisciplinary Graduate School of Engineering Sciences Kyushu University Fukuoka 816‐8580 Japan; ^4^ Department of Research and Innovation Saveetha School of Engineering SIMATS Chennai Tamil Nadu 602105 India; ^5^ Department of Mechanical Engineering Mattu University Mettu 318 Ethiopia; ^6^ Department of Physics Faculty of Science University of Tabuk Tabuk 71491 Saudi Arabia; ^7^ Department of Chemistry College of Science King Khalid University P.O. Box 9004 Abha 62217 Saudi Arabia; ^8^ Nanotechnology Center School of Fashion and Textiles The Hong Kong Polytechnic University Kowloon 999077 Hong Kong; ^9^ School of Chemical Engineering Yeungnam University Gyeongsan 38541 Republic of Korea; ^10^ School of Engineering and Information Technology The University of New South Wales Canberra Australia

**Keywords:** electrode materials, future prospects, MFC challenges, MFC practical applications, microbial fuel cells

## Abstract

Microbial fuel cell (MFC), a clean and promising technology that has the potential to tackle both environmental degradation and the global energy crisis, receives tremendous attention from researchers over recent years. The performance of each system component, including the membrane and electrode utilized in MFCs, has a great effect on the efficiency of converting chemical energy found in organic waste to power generation through bacterial metabolism. The MFCs have diverse applications that are growing day by day in developed countries. This review discusses recently available various potential applications including wastewater treatment, biohydrogen production, hazardous waste removal, generation of bioelectricity, robotics, biosensors, etc. There are still several challenges (e.g., system complexity, economic, commercialization, and other operational factors) for large‐scale practical applications, particularly for relatively low power output and delayed start‐up time, which is also reported in this review article. Moreover, the operational factors (e.g., electrode materials, proton exchange system, substrate, electron transfer mechanism, pH, temperature, external resistance, and shear stress and feed rate) that affect the performance of MFCs, are discussed in detail. To resolve these issues, optimizations of various parameters are also presented. In the previously published studies, this paper indicates that MFCs have demonstrated power densities ranging from 2.44 to 3.31 W m^−^^2^, with Coulombic efficiencies reaching up to 55.6% under optimized conditions. It is also reported that MFCs have achieved the removal efficiency of chemical oxygen demand (COD), total organic carbon (TOC), and antibiotics up to 93.7%, 70%, and 98%, respectively. Finally, this paper highlights the future perspective of MFCs for full‐scale applications.

## Graphical abstract

This review discussed recently available various potential applications of MFCs. There are still several challenges for large‐scale practical applications, particularly for relatively low power output and delayed start‐up time, which is also reported in this review article. Moreover, the operational factors that affect the performance of MFCs, are discussed in detail. Finally, this paper highlights the future perspective of MFCs for full‐scale applications.

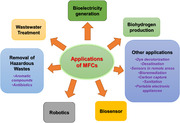

## 1 Introduction

Since fossil fuels have been over‐exploited and consumed over the past century, there has been tremendous environmental pollution, which has greatly facilitated research and development of renewable and ecologically friendly alternative energy.^[^ ^1^, ^2^, ^3^, ^4^, ^5^, ^6^ ^]^ A significant amount of research is being conducted on renewable energy sources to produce power with less emission. Major sources of renewable energy production include hydropower, geothermal, wind, solar radiation, and biomass.^[^ ^7^ ^]^ By 2050, renewable sources are expected to provide 60% of the world's electricity market, and biomass will account for about half of global energy demand and consumption. According to the International Renewable Energy Agency (Irena, 2022), in order for the world to make the necessary changes to ensure the global energy shift from the present scenario to net zero carbon emission scenarios, there is a demand for a rise in both the production and consumption of modern bioenergy. Considering the conventional usage of biomass included, bioenergy presently accounts for the largest share of the world's renewable energy consumption. A significant rise in bioenergy production would be required by 2050 in order to meet the “1.5 °C climate goal scenario”. It may be challenging to achieve this goal without using sustainable biomass for multiple uses, as the amount of bioenergy currently being used is still far less than what is required to accomplish the energy transition. Since the final outcome, when properly developed, will always be more than the sum of its individual components, here is where collaborative and interrelated approaches—which may involve the use of MFCscan really make a difference.^[^ ^8^, ^9^, ^10^ ^]^ The advent of MFCs presents an opportunity to expedite the implementation of decentralized and sustainable energy solutions that can alleviate urban populations' energy limitations and support decarbonization initiatives. The MFC has developed into other uses in addition to energy generation through the use of electrochemically active microorganisms.^[^ ^11^ ^]^ MFCs could play a key role in waste management, reversing the trend of water scarcity, restoring biodiversity, and serving as an alternative energy source to fossil fuels that harm the environment.^[^ ^12^ ^]^ MFCs are a renewable energy technology that can supply fresh water and clean, sustainable energy. Benefits of MFCs include energy production, the use of carbon‐free sources, chemical matter biodegradation, wastewater treatment, bioenergy production, and the removal of contaminants like nitrates.^[^ ^13^ ^]^ By exploiting the unique abilities of microorganisms to produce bioenergy, MFCs have evolved into a ground‐breaking technology in response to the growing worldwide energy demands and environmental concerns.^[^ ^14^ ^]^

MFCs, an environmentally friendly technology for energy conversion, have attracted a great deal of attention. This is due to their exceptional capacity to convert chemical energy found in organic waste into electricity directly while also removing pollutants.^[^ ^1^, ^15^, ^16^ ^]^ MFC is a kind of bio‐electrochemical device that utilizes electroactive microorganisms to convert chemical energy into direct electrical energy using a combination of electrochemical reactions and microbial metabolic from a broad range of substrates, such as wastewater and urine.^[^ ^17^, ^18^, ^19^ ^]^ In 1911, Potter introduced the idea of MFCs for the first time. Anodic and cathodic chambers are found mainly in MFCs, and they are separated by a PEM. A bacteria solution and a substrate are both incorporated into the anode chamber. According to how effectively bacteria can oxidize the substrate, microorganisms are considered. In contrast, an electrolyte solution is found in the cathode chamber to provide oxygen continuously.^[^ ^20^, ^21^ ^]^ Anaerobic bacteria oxidize the material in the anodic chamber of a conventional MFC to produce extracellular electrons, which travel to the cathodic chamber where oxygen captures them, through an external circuit. The electric current is driven by the movement of electrons through the circuit from the anodic to the cathodic chamber.^[^ ^21^, ^22^, ^23^, ^24^ ^]^ Mediator‐less MFC and mediator MFC are two types of MFCs, depending on the need for an external mediator. In mediator MFC, adding synthetic mediators facilitates the movement of electrons from the bacteria to the anode. On the other hand, the mediator‐less MFC can be further categorized based on the microbes' ability to move electrons from the microbial cell to the anode either directly or by self‐synthesized electron shuttles.^[^ ^7^, ^25^, ^26^ ^]^

The performance of MFCs can be enhanced by changing the electrode materials. By enhancing bacterial adherence, electron transport, and modifying the electrode surface, MFCs’ efficiency can be improved.^[^ ^27^ ^]^ Treatment for waste and wastewater with an MFC is efficient because it uses clean energy, operates silently, is extremely efficient, emits low emissions, and offers direct electricity recovery.^[^ ^28^ ^]^ MFC applications appear to demonstrate potential from an economic and environmental perspective.^[^ ^29^ ^]^ Extensive studies were conducted to map the MFC application in various fields like treatment of wastewater, removal of hazardous wastes, bioelectricity generation, biodegradation, biosensor, robotics, etc. Scientific advancements in the electron transport mechanisms and growing recognition of MFCs as renewable and sustainable energy sources prompted these studies.^[^ ^30^ ^]^ MFCs revealed the potential for utilization for efficient treatment of wastewater and simultaneous electricity generation from renewable biomass. Municipal, industrial, and agricultural wastewater can all be treated utilizing MFCs. MFCs were also reported to be helpful for providing low‐power biomedical devices placed in humans with long‐term, consistent power.^[^ ^7^ ^]^

Numerous challenges must be overcome for applications to be efficient in real‐world environments, including pH, temperature, terminal electron acceptors, the anodic chamber's electron transfer mechanism, the substrate, the electrode material, and the membrane. All of these factors can affect the performance of MFCs.^[^ ^28^ ^]^ The challenges associated with scaling up MFC include high fabrication costs, high internal resistance, and material costs. Additionally, lower mixed culture biofilm efficiency on the electrode, the growth of other operational issues over time, and slow pollutant degradation kinetics further complicate the process^[^ ^31^, ^32^, ^33^ ^]^

However, the MFC technologies develop a suitable and environmentally friendly method of producing bioenergy while also treating wastewater. MFCs are more practicable for power generation due to advancements in power densities, COD removal, degradation of pollutants, and a global requirement to generate power except for any CO2 emission.^[^ ^34^, ^35^ ^]^ But the output power achieved by MFC technology is still low, and it can be increased by a few approaches, including proper design, improving electron transfer activity with nanoparticles,^[^ ^36^ ^]^ and employing genetically modified microorganisms.^[^ ^34^ ^]^

Recent developments in MFC technology include several key areas, including deep learning applications, self‐powered biosensors, biomass as fuel, electrode material advances, etc. Nanomaterial‐based electrode research and modification have been essential for increasing power output and cutting costs, which has improved MFC scalability. MFC configuration modification has been the focus of recent research in an effort to enhance power output. Conductive materials, for example, have been demonstrated to improve electron transfer efficiency when integrated into the anode chamber, enhancing the amount of power generated.^[^ ^37^ ^]^ Tsompanas et al.’s study investigated the potential use of neural networks to forecast MFC output, which is directly linked to electrode performance. By offering insights into the electrochemical processes inside MFCs, their findings contribute to the development of more effective electrode materials.^[^ ^38^ ^]^ For environmental monitoring, MFC‐based self‐powered biosensors have become essential devices. As biosensor development moves toward simplification and miniaturization, these devices eliminate the requirement for external power sources. The comprehensive study by Xue et al. examined various sensing techniques in MFC‐based biosensors, highlighting their contribution to the simplicity and miniaturization of biosensor technology.^[^ ^39^ ^]^ It has been considered to employ biomass in MFCs to directly transform chemical energy from organic molecules into electrical energy, offering a viable substitute for conventional fuels. A study by Hao et al. examined the development of self‐powered biosensors using MFCs and enzymatic biofuel cells, which use substrates derived from biomass. These biosensors are appropriate for uses like environmental monitoring and clinical diagnostics because of their benefits, which include ease of miniaturization and simple configuration.^[^ ^40^ ^]^ Intending to maximize performance and integrate into sustainable energy solutions, recent research has used deep learning algorithms to forecast MFC energy production.^[^ ^41^ ^]^ Hess‐Dunlop et al. used long short‐term memory models to predict how much energy soil MFCs would generate. Over a range of timescales, the models' mean average percent error varied from 2.33% to 5.71%, demonstrating the potential of deep learning to improve MFC performance prediction.^[^ ^42^ ^]^

In the previously published review articles, there is still a lack of adequate information regarding MFC with its recent application, challenges, and future prospects. Hence, this review article aims to discuss the most recent application and an overview of MFCs with current information from both industry and academia. In this review study, major applications of MFCs are explored with the most recent relevant information. This paper also presents different MFC challenges that arise in real‐world applications and future prospects of MFC technology.

## 2 Overview of Microbial Fuel Cells

### 2.1 Historical Development of Microbial Fuel Cells

MFC is a bio‐electrochemical system that uses wastewater as the anode feed to produce electricity by degrading organic materials.^[^ ^20^ ^]^ MFC utilizes the catalytic reactions produced by microorganisms to convert the energy contained in chemical reactions in organic compounds into electrical energy.^[^ ^9^, ^24^ ^]^ In the field of producing renewable energy, MFC is one of the less‐explored yet high‐potential areas. The idea of low‐cost power generation is greatly being achieved because of microbes found in biomass.^[^ ^44^ ^]^ Galvani discovered the existence of animal electricity in the mid‐1780s when the nerves of a recently dead frog were connected to long metal wires and pointed toward the sky while a thunderstorm occurred. The frog jumped as if it was alive with each thunderclap. Galvani tried to prove the existence of internal electrical force in living things in the 1780s. Alessandro Volta, who discovered this in the 1800s, strongly disagreed, contending that the external electricity produced by the metal wires is responded to by only animal corpses.^[^ ^45^, ^46^ ^]^

According to Potter, the first instance of bacterial species and electrodes demonstrating electrochemical activity was discovered in the early 20th century, while live cultures of Saccharomyces spp. and *Escherichia coli* (*E. coli*) generated electricity utilizing platinum microelectrodes in a battery‐type configuration with sterile media. In 1931, a voltage of 35 V with a current of 0.2 mA from a stack of bacterial fuel cells was reported by Cohen, which later served as confirmation of this. Although these papers are commonly quoted as the beginning of electro microbiology, it was not until 1963 that a NASA space program showed that it was possible to recycle and transform human waste into electricity during space travels. Although the cell was functional, it was shown to be unstable because of the fluctuating nature of the hydrogen production by the microorganisms. In 1976, Suzuki found a solution to the problems with C. butyricum's hydrogen production and presented a novel idea for MFC design that is still in use today. Later in the 1990s, there was a greater interest in and opportunity for using renewable energy sources as a substitute for fossil fuels in the generation of electricity.^[^ ^20^, ^21^, ^44^, ^47^, ^48^ ^]^

Compared to conventional fuel cells, MFC has few advantages. Instead of using expensive hydrogen or methanol that must be oxidized to produce current in a fuel cell, MFC may convert the current from cheap renewable sources such as biomass and wastewater.^[^ ^49^ ^]^ The utilization of costly and toxic chemical mediators could subsequently be eliminated, revolutionizing the world of MFCs. Currently, numerous nations have started long‐term research initiatives to examine the theory and technology of MFCsin greater detail, ensuring future energy security. The technological development of MFC has been very successful in terms of the biosystem, membrane material, electrode material, and system angle.^[^ ^46^ ^]^

### 2.2 Basic Principle of Microbial Fuel Cells

**Figure 1:**
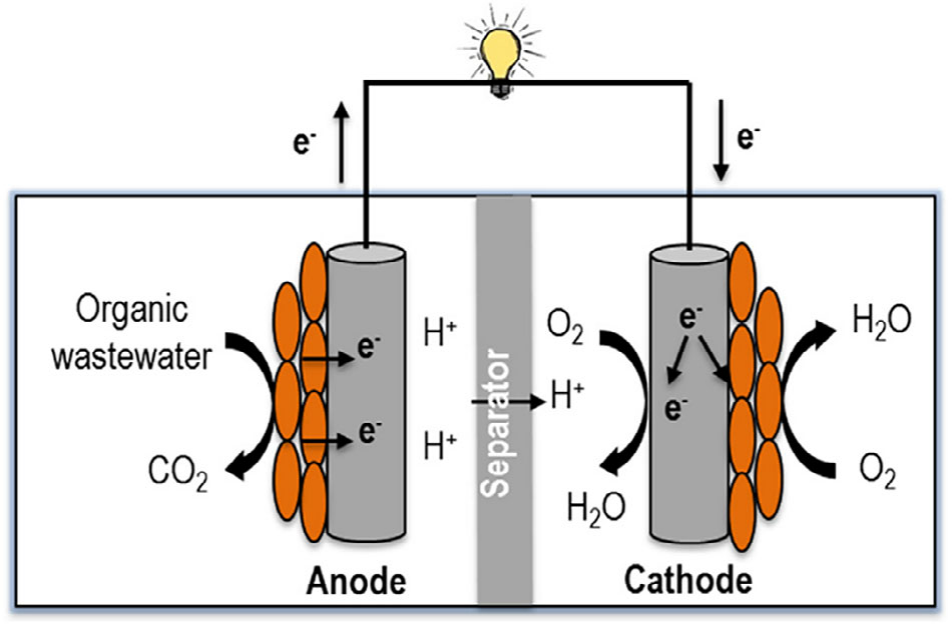
Schematic representation of a typical MFC. Reproduce with permission from ref. [53]. 2018, Elsevier Inc.

An MFC typically consists of an electrolyte, a cathode, and an anode. The cathodic and anodic compartments can be separated by an ion exchange membrane and are connected externally by conducting wire. Microorganisms in a liquid or attached as a biofilm in the anodic chamber oxidize organic substrates to produce protons, electrons, and other metabolites as byproducts. The fundamental MFC makes use of a range of organic substrates, such as alcohols, sugars, acetate, organic compounds, or even wastewater.^[^ ^30^, ^50^, ^51^, ^52^ ^]^ The schematic of an MFC's basic configuration, shown in **Figure** **1**,^[^ ^53^ ^]^ can generally be used to visualize how an MFC functions. Anolyte refers to the collective fluid content of the anode compartment, whereas catholyte refers to the same fluid in the cathode compartment. Wastewater containing organic compounds is the fuel that enters the anode compartment. These organic substrates are oxidized by microbes in the anode compartment, which results in the production of electrons. The electron transport system, which may be an internal component of the microorganisms or an externally added chemical substance known as a “mediator,” is responsible for carrying these electrons to the anode. The electrons travel to the cathode from the anode via an external resistance that is connected externally, providing electrical energy to the associated load along the way. Electric power is produced by the movement of electrons through an external electric circuit. On the other hand, protons produced during the decomposition of organic materials are transferred from the anode to the cathode compartment through the electrolyte. The transferred protons and electrons mix with oxygen to form water at the cathode.^[^ ^28^, ^44^, ^51^, ^54^, ^55^ ^]^ The reactions actually occurring in MFCs can be represented as follows when using acetate as the electron donor and oxygen as the electron acceptor.^[^ ^26^, ^55^ ^]^

(1) $$\mathrm{Anode}\;:CH_{3}\mathrm{COOH}+2H_{2}O\to 2CO_{2}+8H^{+}+8e^{-}$$

(2) $$\mathrm{Cathode}\;:2O_{2}+8H^{+}+8e^{-}\to 4H_{2}O$$

(3) $$\begin{array}{c} \mathrm{Overall}:CH_{3}\mathrm{COOH}+2O_{2}\to 2H_{2}O+\\ 2CO_{2}+\mathrm{Electricity}+\mathrm{Biomass} \end{array}$$

By reducing polarization losses, the power output of MFCs can be improved. These losses include activation losses caused by inadequate contact between the biocatalysts and the electrode surface, ohmic losses due to the system's internal resistance, and concentration polarization resulting from a lack of widely available feedstock.^[^ ^51^ ^]^ The efficiency of MFC systems is also dependent on the “mediated” and “direct” electron transfer mechanisms for electron transfer. The role of “nanowires” and “membrane‐bound cytochromes” in electron transport through direct mode is crucial.^[^ ^30^ ^]^

### 2.3 Materials and Features

**Figure 2:**
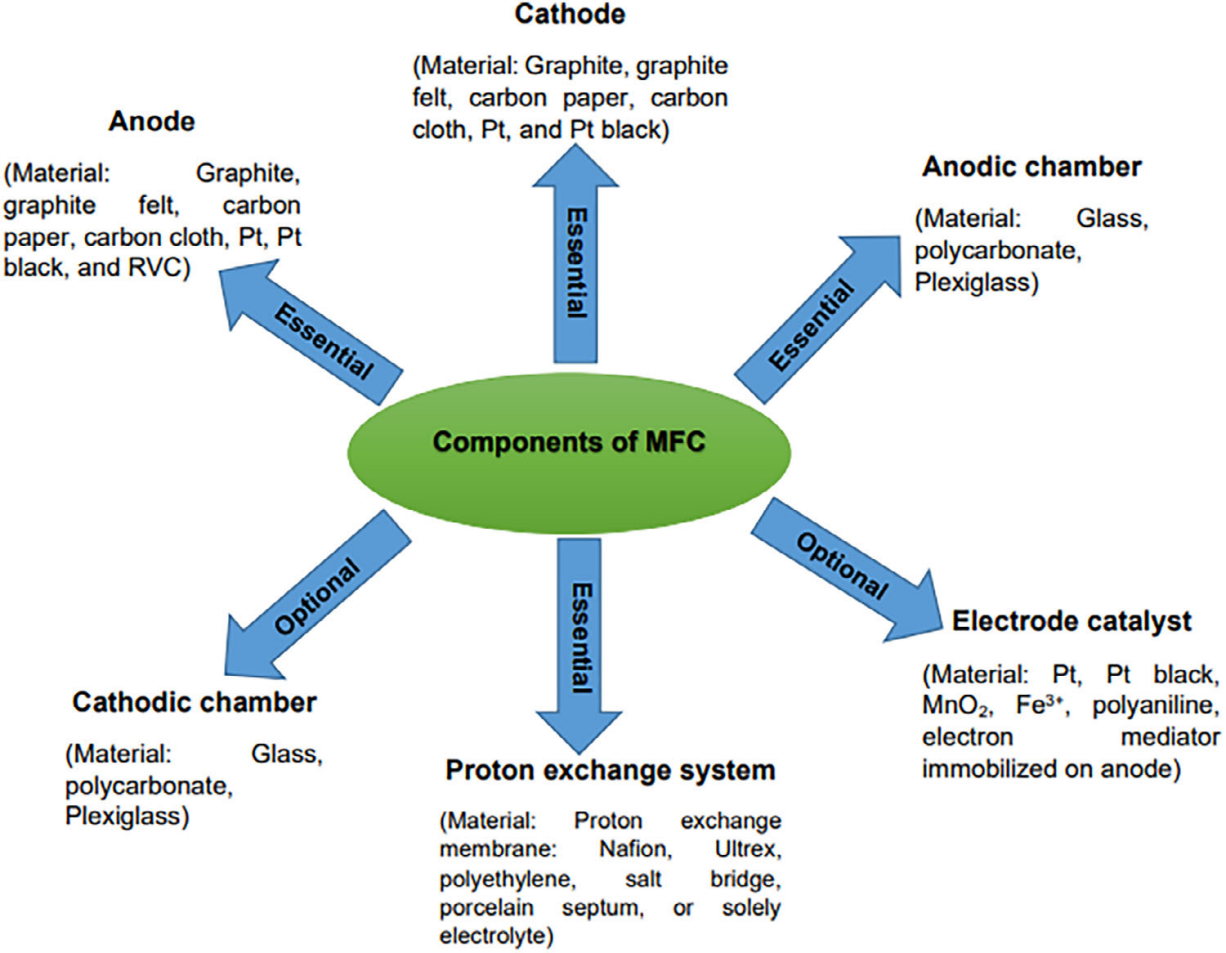
Components and materials used to construct MFCs. Reproduce with permission from ref. [57]. 2017, Taylor & Francis Group.

In terms of bacterial adhesion, electrochemical efficiency, and rate of electron transfer, electrode material performs well for MFCs. The material cost must be minimized and power densities must be increased for MFC technology to be used in a real‐world scenario.^[^ ^21^ ^]^ In order to increase the effectiveness of pollution removal and energy production, electrode materials were studied. The mechanical strength, biocompatibility, chemical stability, and electrical properties of the electrode material should all be extremely acceptable.^[^ ^56^ ^]^ The main components and materials used to construct MFCs are illustrated in **Figure** **2**.^[^ ^57^ ^]^ The materials of the anode and cathode are overviewed below.

#### 2.3.1 Anode Materials

A significant component of an MFC is the anode compartment. The bacteria and anode electrodes serve as electron acceptors, and the substrate is there whether there is a mediator available or not. The electrode material and equipment configuration are a couple of the variables that impact how well MFCs function.^[^ ^21^ ^]^ The optimal anode for MFCs can be constructed from a variety of materials, requiring a large surface area by boosting the extracellular efficiency of electron transfer by a biofilm. However, anodic compounds are also necessary since they improve the ability of anaerobic bacteria to oxidize organic waste by improving their metabolic rates.^[^ ^56^ ^]^ In the anode compartment, electrons are also produced using photosynthetic bacteria and microalgae. In the presence of ideal oxygen and lighting conditions, Chlorella pyrenoidosa donates electrons, resulting in a peak power density of 6030 mW m^−2^.^[^ ^43^ ^]^ The microbial consortia, pH, anode material, mediators, and substrate employed in the anode chamber are specifically regarded as significant factors of MFC performance. It is crucial to select an anode material that possesses a set of strengths, including high physical and chemical stability, low resistance, high conductivity, etc. For MFCs, nonmetallic anode materials that are often utilized include carbon paper, felt, carbon mesh, graphite rods, cloth, carbon brushes, foam, graphite plates, graphite sheets, and reticulated vitreous carbon.^[^ ^58^, ^59^ ^]^ Rod, brush, and veil‐shaped structures can be created using metallic and carbonaceous anodes. Commercially accessible sheets of stainless steel, nickel, and silver can be used as metal anodes.^[^ ^43^ ^]^

**Figure 3:**
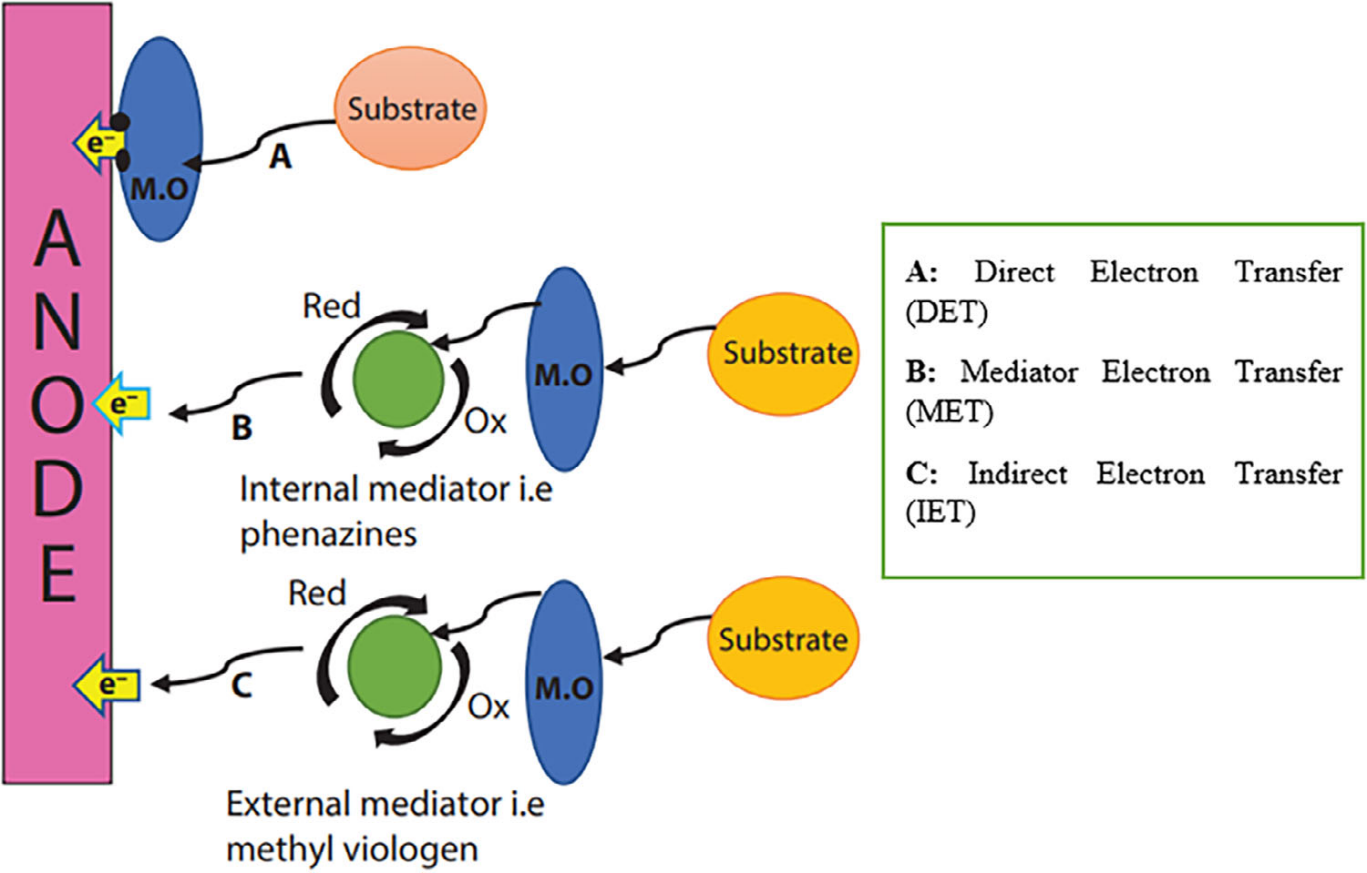
Electron transfer mechanisms in an MFC. Reproduce with permission from ref. [60]. 2021, John Wiley & Sons, Inc.

Microorganisms attached to the anode surface create a biofilm in the anodic compartment that acts as a catalyst for the oxidation process of organic molecules. The electron transfer mechanisms in an MFC are demonstrated in **Figure** **3**.^[^ ^60^ ^]^ Heterotrophic organisms are the most prominent type; they get their power from the oxidation of organic wastes. The electrogenic biofilm S. oneidensis is assumed to be responsible for enabling direct electron transfer (DET) to the anode. While pure cultures of G. sulfurreducens can produce high current densities and produce their own redox mediators, such as phenazines, this process is known as mediator electron transfer (MET). Additionally, with the help of chemical molecule mediators like thionin and Methyl viologen, other pure strains of P. vulgaris, P. aeruginosa, and several types of fermentative bacteria were also employed in MFC. The term of this mechanism is referred to as indirect electron transfer.^[^ ^60^ ^]^

#### 2.3.2 Cathode Materials

The cathode chamber in MFC is considered an electron sink where oxygen is reduced to water. At the interface of the three phases of the cathode chamber—air, liquid, and solid—oxygen is reduced. A catalyst, air diffusion layer, and electrode support make up a typical MFC cathode. Through an external electrical circuit, electrons move from the anode to the aerobic cathode chamber, where they combine with protons on the surface of the cathode electrode.^[^ ^43^, ^61^ ^]^ The cathode chamber is a constraint that has the potential to significantly impact MFC performance. Different electron acceptors are utilized in cathode chambers for cathodic reduction, including ferricyanide, potassium ferricyanide, ferric ions, hexavalent chromium, nitrite, sodium bromated, dichloroethane, fumarate, and nitrate. In single‐chambered MFC, on the other hand, oxygen performs as an electron acceptor molecule. The cathode is another constraining element in the cathode chamber in addition to the electron acceptor molecules. Several materials were utilized as cathodes, including carbon mesh, graphite felt, double‐sided cloth, graphite fiber brush, carbon felt, stainless steel mesh, and carbon cloth.^[^ ^46^, ^58^, ^62^, ^63^ ^]^ The support materials based on cathodic carbon need to be modified with additional catalysts for strong cathodic reactions in MFCs. Platinum is the most commonly utilized cathode catalyst, because of its high efficiency in reducing oxygen.^[^ ^20^, ^61^ ^]^

#### 2.3.3 Membrane

**Figure 4:**
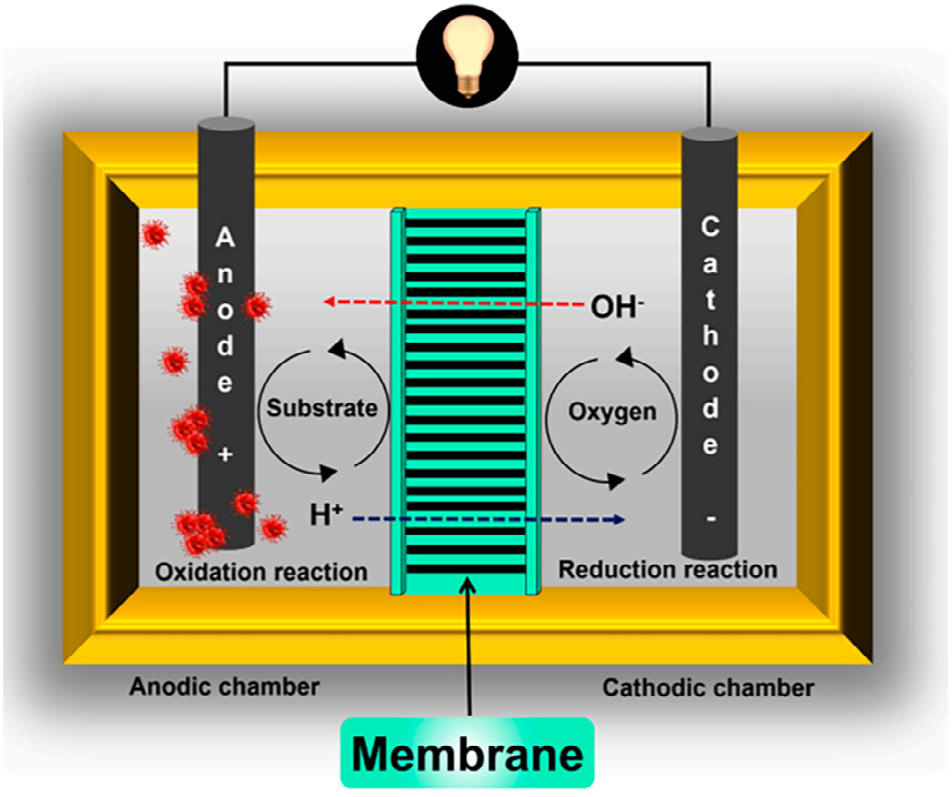
Schematic representation of membrane. Reproduce with permission from ref. [64].

In MFCs, the membrane separator performs a number of crucial tasks. Its aim is to separate the corresponding chemical reactions and avoid short‐circuiting between the electrodes, as was already discussed. On the other hand, protons travel from the anode compartment to the cathode compartment through a membrane separator that is placed between the two chambers. Generally, separators need to have high proton transfer rates, good thermal stability, low gas permeabilities, and biofouling resistance.^[^ ^58^, ^64^, ^65^, ^66^ ^]^ The membrane that separates the anode and cathode chambers enables the recovery of the reaction product from the cathode compartment. Membranes can result in the establishment of a pH gradient and improve electrolyte resistance.^[^ ^31^, ^67^ ^]^ As shown in **Figure** **4**,^[^ ^64^ ^]^ the function of the membrane is to prevent the movement of soluble low molecular‐weight organic molecules from the anode to the cathode. Additionally, preventing the crossing or exchange of microorganisms between chambers is another function of the membranes used in MFCs that is poorly discussed. In an MFC, a membrane's performance will often be primarily determined by its transport properties.^[^ ^64^ ^]^ Excellent proton transfer efficiency and selectivity are required of a suitable and optimal separator/membrane to prevent the unintended transport of other molecules. Proton conductivity and selectivity, ion exchange capacity (IEC), membrane hydrophilicity, oxygen permeability, thickness, resistance, and other factors are taken into account while selecting separators or membranes.^[^ ^1^, ^18^, ^65^ ^]^

In MFC systems, the membrane surface also affects the performance of the membrane and power generation. Currently, it has been reported that a variety of membrane materials serve as separators in MFC systems. Membrane materials commercially include, among others, Nafion, Zirfon, and Ultrex.^[^ ^68^ ^]^ The properties of typical membranes/separators used in MFCs are shown in **Table** **1**.

**Table 1:** Properties of typical membranes/separators utilized in MFCs.

Membrane/Separator	IEC [meq g^−1^]	Proton conductivity [×10^−2^ S cm^−1^]	Water uptake [%]	Thickness [cm]	Refs.
Polyvinyl alcohol–Nafion–borosilicate	–	7	105	0.0167–0.0184	[69]
Sulfonated‐oxy‐polybenzimidazole	1	7.83	29	0.002–0.003	[70]
SPEEK/7.5% TiO2	1.98	0.187	21.83	0.018 – 0.002	[71]
Nafion 117	1.23	0.2	22	0.019	[72]
SPSEBS	–	38.2	164	0.018	[73]
SPAEK/PW–mGO 1 wt.%	2.51	26.07	156	0.004–0.005	[74]

### 2.4 Classifications of Microbial Fuel Cells

MFC is typically divided into two types depending on the transfer of electrons from bacteria to the anode. Mediator and mediator‐less are two forms of MFCs available based on the need for an external mediator.^[^ ^7^, ^49^ ^]^ Active mediators are utilized in mediator‐based MFCs to transfer electrons during electrochemical processes. One such substance, methylene blue, has been employed as an active mediator in MFC technology to improve power output. The same process as for mediator‐based MFCs is employed for non‐mediated MFCs, but instead of using expensive chemicals as mediators, bacteria are used. Electron carriers in this scenario include proteins, microbial pili, and electro‐active bacteria.^[^ ^28^, ^75^, ^76^ ^]^ On the way of the technology's development, several MFC types and configurations can be observed that aim to enhance the performance of MFCs. In contrast to mediated and non‐mediated, MFCs can also be assigned several common names depending on the operational conditions, systems, and fundamental sources upon which they are established. For example, double, single, and multi‐chambered MFCs, as well as those based on soil, mud, plants, sediments, wetlands, sediments, and many others, are given specific names in accordance with their configuration. In MFCs with double chambers, one bottle acts as the anode while the other, which is separated by PEM, acts as the cathode. The anode and cathode are both contained in the same chamber in a single chambered MFCs. The power output of MFCs is directly impacted by this classification, making it more significant.^[^ ^62^, ^75^, ^77^, ^78^ ^]^

### 2.5 Performance Evaluation of Microbial Fuel Cell (MFC)

The completion of a circuit fulfills the requirement for MFCs to function effectively. The microorganisms start working on oxidizing and reducing the organic “fuel” as soon as it enters the anode compartment to establish the life‐sustaining adenosine triphosphate (ATP) that fuels their cellular functions. As byproducts, the anode acts as the electron acceptor inside the bacteria's electron transport system, producing electrons, protons, and carbon dioxide.^[^ ^79^ ^]^ The voltage and current measured across the external load are used in an MFC to calculate power as follows:

(4) P=IU

In an MFC, the current can be obtained by measuring the voltage drop across the resistor of the external circuit using *I*  =  *U*/*R*_ex_, thus the power can be represented as a function of *U* and *R*_ex_:^[^ ^80^ ^]^

(5) $$P=\frac{U^{2}}{R_{\mathrm{ex}}}$$

Power can also be represented in terms of calculated current as:

(6) $$P=I^{2}\;R_{\mathrm{ex}}$$

Electrical parameters such as power density, internal resistance, current density, biodegradation efficiency, and potential difference are commonly used to evaluate the performance and efficiency of MFCs. An interesting factor that combines the two earlier ones is coulombic efficiency (CE): the number of electrons used to feed the current obtained from the oxidation of organic substrates.^[^ ^7^ ^]^ Presenting MFC performance is now typically done using polarization (voltage vs current) curves. This shows the maximum current and open‐circuit voltage of the cell in addition to the voltage–current behavior characteristics that provide the most reliable information about internal resistances.^[^ ^55^ ^]^ One of the most crucial factors in assessing the performance of an MFC is power density. In order to compare different MFC systems, power output is usually standardized to projected electrode surface area, also referred to as surface power density. The reason that maximum current density is rarely mentioned in MFC research is likely because the main objective of these studies is to enhance the power output, even if the power density of MFCs is low at maximum current density. However, while assessing the electrode materials and the bacterial electrochemical capabilities, the maximum current density is a crucial factor.^[^ ^81^, ^82^ ^]^ The evaluation of the electron recovery as current from the organic compounds is performed using CE. When comparing the performance of MFCs with more conventional energy conversion techniques like combustion, energy efficiency is an extremely relevant factor.^[^ ^81^, ^83^ ^]^

## 3 Applications of Microbial Fuel Cells (MFCs)

**Figure 5:**
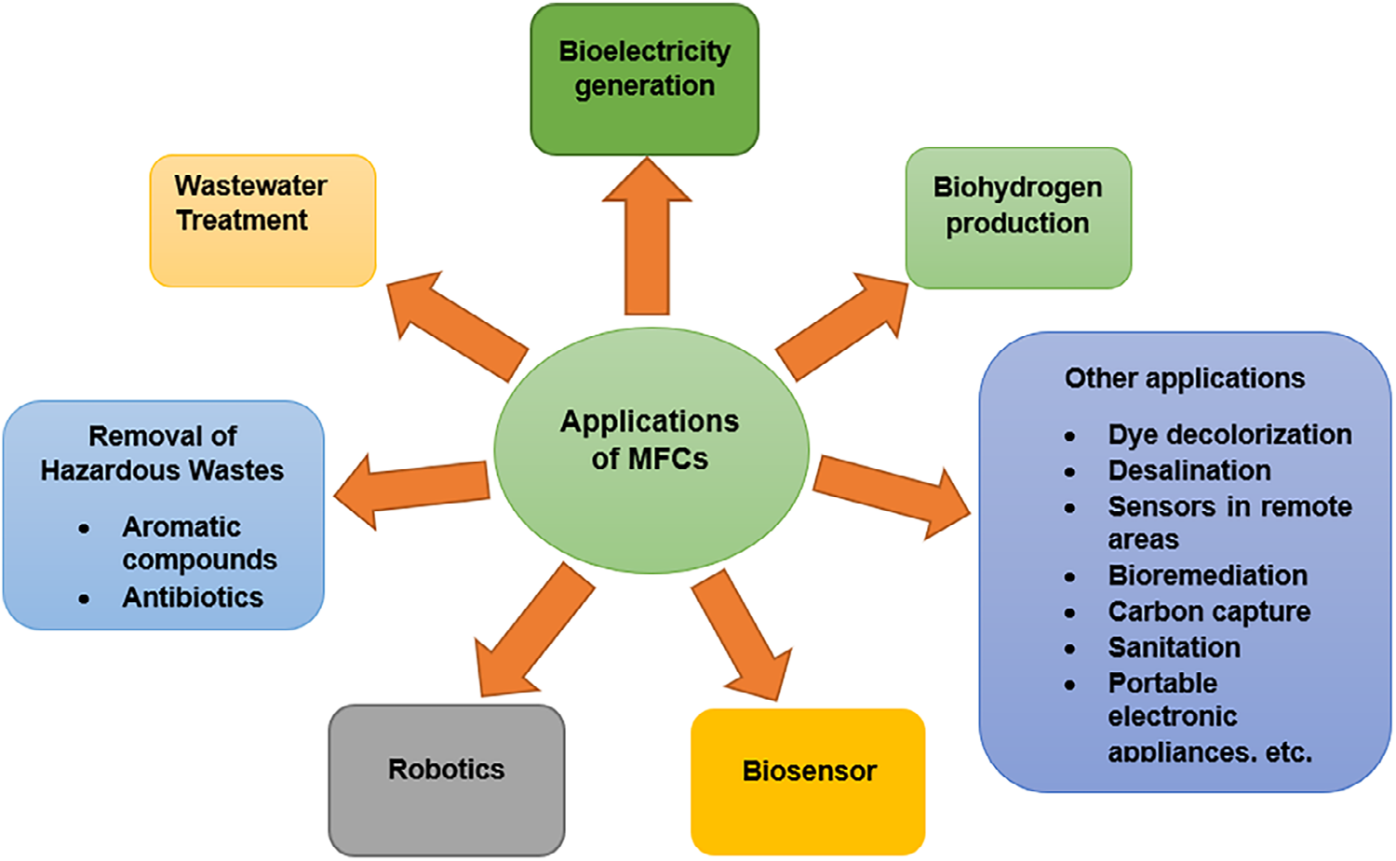
Schematic representation of various applications of MFCs.

If applied correctly and on a large scale, MFC technology has a variety of uses. Production of power, biohydrogen generation, wastewater treatment, biosensors, removal of hazardous wastes, and robotics applications are some of the major applications of MFCs.^[^ ^75^ ^]^ There are also numerous applications of MFCs over the world. In this study, the most recent and major applications have been presented. **Figure** **5** demonstrates the schematic representation of different applications of MFCs. The major applications of MFCs are discussed below.

### 3.1 Generation of Bioelectricity

**Figure 6:**
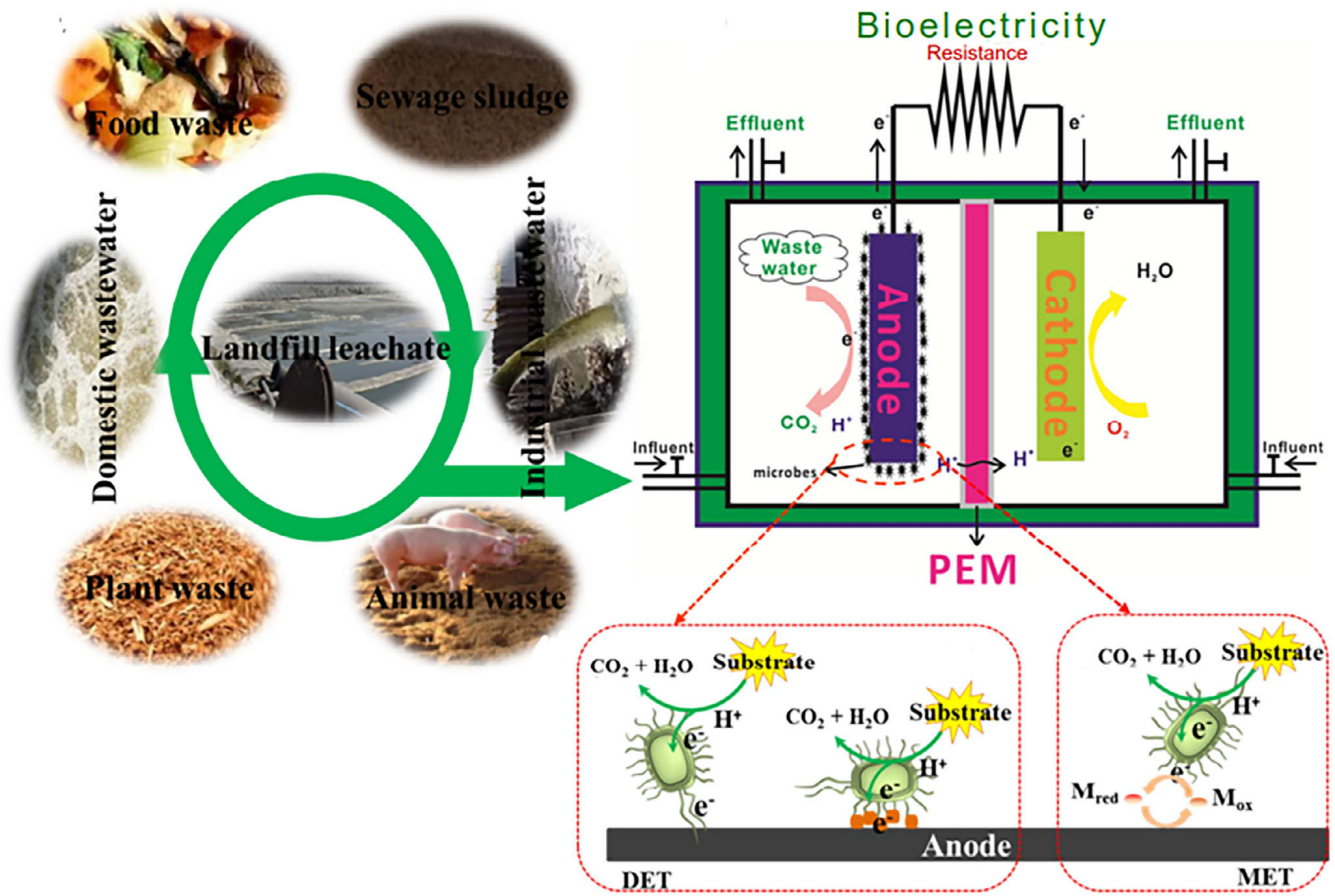
Schematic representation of bioelectricity production process in MFC. Reproduce with permission from ref. [84]. 2020, Elsevier Ltd.

In the current energy crisis, the primary application and output of MFCs is electricity production. Kumar et al. demonstrated how this MFC application reported power production using exoelectrogens. The study found that MFC technology is the best and most eco‐friendly method among all electricity‐producing technologies. It also discussed bacterial activity, efficiency, and the system's functioning mechanism.^[^ ^75^ ^]^ Utilizing microorganisms, MFCs can convert the chemical energy contained in the chemical compounds of biomass into electrical energy.^[^ ^57^ ^]^ The schematic representation of the bioelectricity production process is shown in **Figure** **6**.^[^ ^84^ ^]^ It was demonstrated that a two‐chamber MFC can generate electricity utilizing glucose as the electrolyte and isolate the cathode and anode using a PEM. At the electrode surfaces, the maximum power of 3.6 W m^−2^ was achieved. No methane production was seen while the MFC was producing energy; instead, the bacteria were helping to transmit electrons to the electrode.

In the double chamber MFC, the graphite electrode and glucose were produced as an electrolyte.^[^ ^85^, ^86^ ^]^ By removing a membrane from a double‐chamber MFC, a single‐chamber MFC was developed. Using sediment from river water, a single chamber MFC was developed without the use of PEM.^[^ ^87^ ^]^ The MFC used carbon fabric electrodes to generate an OCV of 0.43 V. The anodic charge transfer resistance increases in MFC with more oxygen, finally limiting the density of power at the maximum current rate. For the purpose of generating energy from polyalcohols using carbon cloth electrodes, a single‐chamber MFC was developed.^[^ ^88^, ^89^ ^]^ Bond and Lovley^[^ ^86^ ^]^ noted that R. ferrireducens was able to generate electricity with an electron yield of 80% because chemical energy produced by the oxidation process is converted directly into electricity instead of heat. The wastewater COD level and the resistance utilized in an MFC determine how much electricity is produced.^[^ ^43^ ^]^ The world's MFC experts have implemented a number of innovative efforts to improve fuel cells' power production. Many of them are developing brand‐new MFC designs that operate MFCs under particular circumstances and utilize various efficient materials for the electrodes and membrane.^[^ ^90^ ^]^

### 3.2 Biohydrogen Production

MFCs have been investigated for producing biohydrogen in addition to electricity, providing a sustainable method of energy production. The invention of microbial electrolysis cells (MECs), which improve the efficiency of hydrogen production when combined with MFCs, is an important development in this field.^[^ ^91^ ^]^ MEC is a recent innovation in hydrogen production. In two chambered fuel cells that were separated by Nafion 117, the acetate and domestic wastewater served as the substrate. It was discovered that 2.9 mol‐H_2_ was produced for every mole of acetate after a small amount of voltage was supplied to the circuit. Depending on the voltage supplied, the hydrogen's CEranged from 60 to 78%. More than 90% of the electrons were recovered as hydrogen.^[^ ^92^ ^]^ MFC is easily modified to produce hydrogen by simply cutting off the oxygen supply and applying a small voltage to lower the protons in the cathodic compartment.^[^ ^44^ ^]^ It is thermodynamically unfavorable to produce hydrogen in an MFC from the protons and electrons produced by microbial metabolism.^[^ ^57^ ^]^ Electric current is supplied at the cathode to complete the reaction between the electron and proton. The electrical requirement can typically be satisfied with >0.3 V. In the MFCs, it is simple to obtain such low voltages. Hence, the MFCs can be combined with MEC to provide electricity and electrical requirements. The hydrogen generated by the MEC can be simply stored and then used to generate electricity.^[^ ^62^ ^]^

The thermodynamic barrier was overcome by Liu et al.^[^ ^93^ ^]^ by applying an external potential to boost the cathode potential in an MFC circuit. In this method, hydrogen is produced at the cathode when protons and electrons from the anodic reaction combine. Since oxygen is not required in the cathodic chamber during the production of biohydrogen utilizing MFCs, oxygen leakage into the anodic chamber is not a reason to be concerned. To overcome the inherent low‐power properties of MFCs, hydrogen can also be collected and stored for future usage.^[^ ^57^ ^]^ In order to achieve high‐rate biohydrogen production, Qing et al.^[^ ^94^ ^]^ found that modifying the cathode with an iron‐sulfide (FeS) catalyst in a single‐chamber MEC greatly enhanced the hydrogen evolution reaction. Another study by Albuquerque et al.^[^ ^95^ ^]^ utilized rice straws that had been prepared with sodium hydroxide in MECs to produce hydrogen efficiently by combining fermentation and saccharification processes. According to studies, MEC configurations that feature a single chamber and no membranes can lower internal resistance and improve the rate at which hydrogen is produced. Furthermore, it has been found that applying external voltages to MECs enhances the efficiency of hydrogen production, emphasizing the significance of operational parameters. In their study, Wenzel et al.^[^ ^96^ ^]^ found that combining an MFC with a biohydrogen reactor successfully raised the energy yield from cheese whey, an organic matter‐rich by‐product of the dairy industry. The initial conversion of cheese whey into hydrogen in this integrated system was accomplished by dark fermentation, which resulted in an effluent that was high in volatile fatty acids. When this effluent was used as a substrate for the MFC, the maximum power density achieved was 439 mW m^−2^, which was much higher when raw cheese whey was used directly. The potential of coupling dark fermentation with MFCs to improve energy recovery from waste substrates is highlighted in this work. A study by Chandra et al.^[^ ^97^ ^]^ focused on recovering bioenergy from jackfruit waste by dark fermentation and then used stacked MFCs to generate power. Using this approach, Enterobacter aerogenes produced biohydrogen from waste jackfruit peels that were high in carbohydrates, yielding 5.02 mol H₂/kg COD_ae_d. A maximum power density of 13.69 W m^−^^3^ was achieved by using the remaining fermentation medium in MFCs that had been inoculated with Pseudomonas aeruginosa. The power density increased by 27.9% when MFCs were operated in parallel stacks, highlighting the benefits of stacking designs for improved energy production. The feasibility of coupling dark fermentation with stacked MFCs to enhance the generation of bioenergy from agricultural waste is demonstrated by this study. Another investigation by Samrot et al.^[^ ^98^ ^]^ examined the use of MFCs to produce biohydrogen from wastewater from rice mills. The study showed that the organic‐rich wastewater from rice mills can be used as a good substrate for the synthesis of biohydrogen, emphasizing the possibility of integrating waste management with the production of renewable energy.

### 3.3 Removal of Hazardous Wastes

#### 3.3.1 Aromatic Compounds

A class of hazardous or problematic breakdown organic contaminants includes aromatic compounds and related compounds. Aromatic compounds are common pollutants that the pharmaceutical industry releases. PWW, which contains numerous hazardous recalcitrant chemicals and high salinity, must thus undergo special treatment before being released into the environment.^[^ ^99^ ^]^ In one experiment, PWW including acetone or phenol was detoxified and generated energy at the same time utilizing a two‐chambered MFC. The MFC had an outstanding contaminant removal rate with no acetone or phenol identified when the amount of phenol was not greater than 50 mg L^−1^ and the amount of acetone was not greater than 100 mg L^−1^.^[^ ^100^ ^]^ Because of its recovery of energy and the anodic biofilm's catabolic flexibility, the MFC can remove aromatic pollutants from PWW.^[^ ^101^ ^]^

#### 3.3.2 Antibiotics

Pharmaceutical companies, hospitals, and the animal breeding industry all release massive amounts of antibiotics into the environment, yet only a few water treatment plants follow the rules, resulting in the environmental release of leftover antibiotics. Environmental degradation has increased as a result of antibiotic abuse and antibiotic leftover in natural systems. Traditional physical, chemical, and biological techniques to remove antibiotics from sewage water are energy and chemically expensive, and biological processes are ineffective at breaking down antibiotics. MFCs have recently been utilized to break down refractory organic compounds.^[^ ^99^, ^102^ ^]^ A two‐chambered MFC was utilized in one experiment to examine the breakdown of metronidazole and evaluate how antibiotics affected the effectiveness of energy production. It was found that MFCs, which have been used for treating contaminated water, were used to break down antibiotics like metronidazole.^[^ ^103^ ^]^

### 3.4 Wastewater Treatment

**Figure 7:**
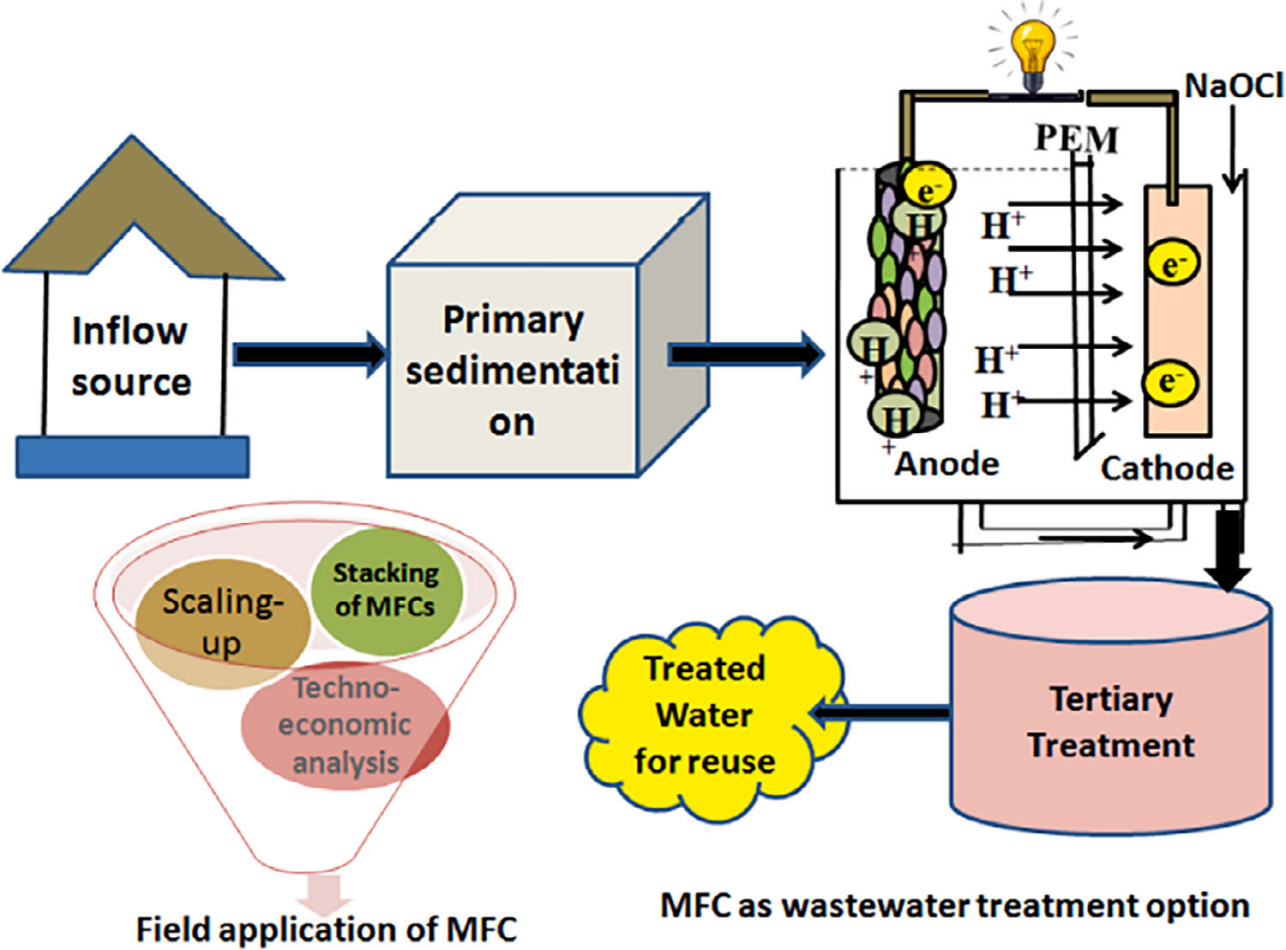
Domestic/industrial wastewater treatment process using MFC. Reproduce with permission from ref. [104]. 2021, Elsevier Ltd.

Wastewater treatment utilizing MFCs commenced in 1991. Various chemical compounds in wastewater from municipalities can be used to power MFCs. The amount of electricity produced by MFCs during wastewater treatment can be reduced by half compared to conventional treatment methods. Traditional processes require a large amount of electricity to acidify the activated sludge.^[^ ^57^ ^]^ **Figure** **7** illustrates a wastewater treatment process utilizing MFC.^[^ ^104^ ^]^ A wastewater treatment plant consists of a number of procedures that aim to treat wastewater effectively and affordably while also keeping track of the treated effluent's pollution load. Domestic wastewater treatment plants comprise screens and grit chambers; trickling filters; biological treatment steps with activated sludge treatment; primary clarifiers; etc. Regarding the biological treatment of wastewater, it is technically responsible for removing a significant amount of organic matter and nutrients as well. From the viewpoints of reducing pollutants, lowering costs, producing bioelectricity, and system sustainability, the MFC potential for wastewater treatment was studied.^[^ ^30^, ^35^ ^]^ MFCs are cutting‐edge, environmentally friendly wastewater treatment systems that utilize wastewater as a substrate and accordingly generate energy while also generating other products with added value. Researchers from all over the world are focusing on various MFC designs using different substrates. They have discovered various findings related to CE, COD removal, the effect of substrate concentration on loading rate, and increasing power density.^[^ ^43^ ^]^ **Table** **2** provides some examples of MFC performance in treating wastewater.

**Table 2:** MFC performance for wastewater treatment.^[^ ^62^ ^]^

MFC type	Wastewater	% COD removal	Refs.
Single‐chamber	Olive mill wastewater	65	[105]
	Cadmium	90	[106]
	Azo dye Congo red	98	[107]
	Chromium (VI)	99	[108]
	Biodiesel wastes	90	[109]
Double‐chamber	Chemical wastewater	63	[110]
	Domestic wastewater	88	[111]
	Real urban wastewater	70	[112]
	Food waste leachate	85	[113]

Although MFCs are unable to entirely treat extremely toxic wastewater, they can significantly lower the COD of the wastewater and fulfill discharge requirements before they are released into the environment. The MFCs have demonstrated up to 98% COD removal from wastewater.^[^ ^62^ ^]^ During the treatment of wastewater, MFCs can promote the advancement of bio‐electrochemically active microorganisms. Due to concerns about scaling up, single‐compartment, continuous flow, and membrane‐less MFCs are preferred for wastewater treatment.^[^ ^25^ ^]^ Large‐scale industries can use this technology to treat their wastewater before discharging it into the environment.^[^ ^114^ ^]^

### 3.5 Biosensor

A promising biosensor system has been identified as the MFC. MFC‐based biosensors may detect a variety of conditions in liquid samples, including toxicity and biochemical oxygen demand (BOD), depending on the biological activity affecting the current flow of MFC. BOD biosensors based on MFC rely on a positive linear correlation between the BOD value and the production of electric current within a particular range in order to function.^[^ ^115^ ^]^ Utilizing MFC technology as a sensor for contaminant detection and in situ procedure control and monitoring is another potential use for the technology.^[^ ^57^ ^]^ A biosensor is an analytical tool combining the physicochemical detector with a biological element to detect analytes.^[^ ^43^, ^116^ ^]^ The use of MFC as a sensor, which is highly beneficial in the treatment of wastewater and processing of portable water, makes it simple to detect and monitor the toxicants present in the water.^[^ ^117^ ^]^ Using MFC as sensors, it is not only possible to detect wastewater toxicity in real‐time, but also acidic toxicity, surfactant toxicity, and formaldehyde toxicity.^[^ ^118^ ^]^ The MFC has been reported to be a useful biosensor for identifying pollutants and organic compounds in wastewater. Exoelectrogens in the anode chamber of the MFC‐based biosensor act as a signal generator while electrodes and PEM serve as the transducer. The MFC biosensor's main benefit is its long‐term stability. Hence, exoelectrogenic biofilms reduce the need for the replacement of sensing elements and increase the lifespan of sensing elements.^[^ ^90^ ^]^ A biosensor based on self‐powered MFC for the measurement of toxicity levels of heavy metal in wastewater was developed by Dengbin et al.^[^ ^119^ ^]^ The experimental design was used to evaluate six heavy metal ions at a concentration of 2 mg L^−1^. The results demonstrate that MFCs can be used as biosensors as well. The initial study to ensure the availability of MFC‐based biosensors to measure wastewater BOD concentration was conducted by Karube et al.^[^ ^120^ ^]^ Several MFC‐based biosensors for BOD monitoring have been developed recently. The biosensor based on MFC was developed by Commault et al.^[^ ^121^ ^]^ with biofilms dominated by Geobacter to measure the BOD of milk over 17.5 h. When compared to the traditional BOD5 method, this biosensor may attain reproducibility of 94% while producing only 7.4% error during milk BOD detection. It suggests that MFC‐based biosensors have the ability to quickly and precisely determine the BOD of dairy wastewater.

### 3.6 Robotics

Robots can be powered by MFCs, which can generate bioelectricity from energy trapped in both complicated wastewater and pure compounds. Robots of various generations that use MFCs as their power sources have been developed and reported.^[^ ^51^ ^]^ Gastrobot is a class of intelligent bio‐electrochemical robots that get their energy from the microbes that help real food be digested. Wilkinson's Gastronome, which utilized a chemical fuel cell and bank of Ni‐Cd batteries, was the first robot powered by bacteria in the world.^[^ ^51^, ^122^ ^]^ Another type of autonomous robot is the EcoBot, which uses MFC designed for food waste and insects as its internal power source.^[^ ^123^ ^]^ The EcoBot project has produced three robots: EcoBot‐I, EcoBot‐II, and EcoBot‐III. They are all based on the same basic principle.^[^ ^124^ ^]^ As the first robot, EcoBot‐I emerged in the early 21st century, powered by an MFC that used sugar as its only substrate, even though many robots had been developed over the years employing MFC.^[^ ^44^ ^]^ In order to produce higher energy and improve stability, EcoBot‐II was developed in a process where refined biomass in the form of an insect is turned into usable power. The actuation, sensing, and communication tasks were performed by these robots.^[^ ^123^ ^]^

According to Ieropoulos et al.’s study,^[^ ^125^ ^]^ the idea of incorporating MFCs into robots has developed over the past century, with significant developments in the last several decades. Through this integration, an artificial symbiosis is made possible, in which robots use MFCs to break down organic materials and provide electricity for their operations. Greenman et al.^[^ ^126^ ^]^ developed MFC‐based thermosensors for robotic applications in another study. These sensors provide a sustainable and self‐powered sensing mechanism for robots that operate under different thermal conditions by using the metabolic process of microbes to identify variations in temperature. By converting waste into useful energy, MFC integration into robotics not only improves energy autonomy but also advances environmental sustainability. This strategy is in line with the advancement of self‐sufficient robotic systems and the increasing interest in renewable energy sources. The sub‐milliliter MFCs developed by Philamore et al.^[^ ^127^ ^]^ were designed to power soft robotics. These small MFCs can be incorporated into soft robots' flexible frames to provide a steady, biodegradable energy supply. The development of environmentally friendly and self‐sufficient robotic systems aligns with this strategy. According to research by Greenman et al.,^[^ ^128^ ^]^ MFCs can produce more power by optimizing their electrode substances, reactor designs, and stack configurations. This provides them with a more feasible option for powering robotic systems. MFC technology's scalability is essential for real‐world robotic applications. Calignano et al.^[^ ^129^ ^]^ investigated the additive manufacturing (3D printing) of MFCs in detail with the goal of developing small reliable energy sources for robotic applications. In order to develop more efficient and energy‐autonomous robotic systems, the study showed that it is possible to integrate MFCs directly into robotic architectures. Despite these developments, there are still issues with maximizing MFCs' scalability and efficiency for real‐world robotic applications. The goal of ongoing research is to fully utilize MFCs' potential in robotics by enhancing power output, miniaturization, and combining strategies.

### 3.7 Other Applications of MFCs

The most useful applications of MFCs have been illustrated in Figure 5. In this section, miscellaneous applications of MFCs are discussed with current information. Along with the current generation, the performance of MFCs for azo dye decolorization was also explored. In the air–cathode MFC, glucose and Congo red were enhanced. The current generation is considerably impacted by this enrichment process while the decolorization of dye is never affected. After 170 h of operation, the MFC's decolorization was found to be 90% effective, and its power density was 192 mW m^−2^, with a 350 mV cell voltage. According to studies, the electro‐Fenton dye decolorization process in MFC is the most effective way to handle colors since it is both energy‐efficient and cost‐effective.^[^ ^92^ ^]^

Seawater or brackish water desalination consumes a significant amount of energy and has considerable operational costs. To increase the efficiency of desalination, a hybrid design combining microbial desalination cells, microbial osmotic fuel cells, and forward osmosis membranes has recently been developed. The desalination process has been enhanced by this coupled system, which also produces a significant amount of energy (0.16 kWh m^−3^).^[^ ^92^ ^]^ Robots inspired by biology, radio devices, portable electronics (such as cellphones, coffee makers, TVs, laptops, and so on), and even low‐energy lighting devices can all be powered by the electricity produced by MFCs.^[^ ^130^ ^]^

It has become crucial to continuously monitor our environment in order to recognize the harmful effects of pollution, such as global warming and climate change. As a result, sensors are being placed in numerous places for monitoring ecosystems, weather forecasting, agricultural uses, fisheries investigations, etc. The biggest challenge in using sensors in remote areas is providing them with continuous electricity for a long period of time. MFCs would be the better substitute source to power the sensors because they can run for a long period on local resources. Sediment MFCs have shown great potential as an innovative energy‐harvesting device that provides reliable, maintenance‐free power for a long time. This extended power supply can increase the lifespan of communication systems and sensors. Installations of sediment MFCs are placed in rivers and lakes where the sediments located at the bottom of those rivers and lakes serve as a renewable fuel source for the MFCs.^[^ ^77^, ^131^, ^132^ ^]^

The process of bioremediation includes the transfer of electrons from an electrode to a chemical species. Chemicals were minimized in the cathode. However, it might be able to carry out chemical oxidation at the anode. The biodegradation of petroleum molecules produces long‐lasting power. MFC may be utilized to improve the bioremediation of petroleum‐contaminated groundwater in anaerobic environments. It is extremely challenging to remove heavy metals from wastewater using a conventional removal technique. The designs of MFC have a great deal of potential for eliminating heavy metals from wastewater pollution.^[^ ^43^, ^133^ ^]^

A photosynthetic MFC has the capacity to capture CO_2_ emissions and release useful O_2_ gas into the atmosphere. At the anode, CO2 gas is released, and it travels to the cathodic chamber, where there are numerous populations of bacteria. MFC is a promising technology that can generate zero CO_2_ emissions, raising hopes for future fuel cell‐based electricity.^[^ ^43^ ^]^

MFC field application can be a feasible and realistic solution for sanitation if septic tank systems are updated with cutting‐edge bio‐electrochemical technologies. This recently developed bio‐electrochemical technology has the potential to be a practical substitute for efficient and sustainable sanitation as well as bioenergy recovery. Such technology may efficiently remove organic matter, sulfur, and nutrients, treat wastewater for disinfection, recover valuable resources, eliminate odors, and generate power for LED lights and other electronic appliances.^[^ ^134^ ^]^

## 4 Challenges of Microbial Fuel Cells

The MFC performance is primarily influenced by the electrode material, wastewater properties, design elements, operating aspects, inoculum conditions, operating aspects, and other parameters. The multidisciplinary process of scaling up this technology involves the study of concerns in the fields of applied engineering, environmental engineering, electrochemistry, biotechnology, economics, and material science.^[^ ^134^ ^]^ Therefore, there are a variety of factors that limit the practical application and scaling up of MFCs, some of which are discussed below.

### 4.1 Challenges to Large‐Scale Applications

The application of MFC technology in larger‐scale power production is now unfeasible due to a number of serious challenges. First, the MFC output power is still not enough for practical uses to power the sensing devices. The power performance of MFCs has been widely studied, focusing on MFC structures, materials, electrochemically active bacteria, and operational conditions. However, the results are not significant compared to other power technologies. Second, the amount of power produced by MFCs does not increase linearly with scale. The MFCs' output power and wastewater treatment capacity do not improve correspondingly even if they can be manufactured for large‐scale applications.^[^ ^135^ ^]^ Despite numerous developments, there are still many challenges to overcome before MFC technology can be widely used for power generation. This is primarily due to the comparatively low power output in large‐scale systems. This prevents large‐scale WWT from scaling up, necessitating the use of more reactors with smaller individual capacities. The amount of improvement in power outputs also will definitely determine the practical application realization and the success of MFCs in the future on a larger scale. The expected output power for the upscaled MFCs is at least 400 W m^−3^ and the current density is ≈1500 A m^−3^.^[^ ^136^ ^]^ The large‐scale MFC practical applications are still greatly limited by their low power output and high operating costs. In large‐scale MFC applications, the composite anode may provide an additional option to satisfy this requirement.^[^ ^59^ ^]^ There are also several limitations to large‐scale applications of MFCs, which have been explained in the below categories.

#### 4.1.1 Economic Challenges

Modernization of new MFC designs has been prompted by a significant increase in power production as well as a decrease in overall capital investment to achieve a safe cost‐benefit ratio.^[^ ^134^ ^]^ The deployment of MFC at the laboratory scale is constrained by low output power and economic factors. These constraints indicate the need for finding low‐cost substitutes for catalysts, electrode materials, and membranes as well as the utilization of power distribution devices for the storage of electrical energy in order to keep it suitable with current technology. The techno‐economic performance of MFC technology can be evaluated to comprehend its impact on commercialization planning. Economic aspects like the capital costs of the reactor include the cost of the membrane, electrode, reactor manufacture, coatings used on any electrode, reactor maintenance, etc. MFCs have an extremely high capital cost that is usually even 30 times higher, in comparison to traditional wastewater treatments. The challenges in cost and operation have restricted the development of MFCs, yet attempts have been made to run MFCs at lab scales to assess the viability of MFC operation at large scales to move MFCs from lab scale to large scale.^[^ ^104^ ^]^ However, numerous challenges should be addressed before realizing the potential of MFCs in the global market.^[^ ^134^ ^]^

Economic issues that must be resolved to enable MFCs to be broadly commercialized. In order to address the high cost of electrode materials used in MFCs, researchers are seeking more affordable alternatives such as carbon‐based electrodes and biocatalysts, which can lower total costs.^[^ ^137^ ^]^ Investigating reactor design and process optimization innovations is necessary to maintain MFC efficiency and lower costs on a larger scale. The initial expenditures of MFC infrastructures may be outweighed by energy recovery and operational cost reduction. Improving reliability and lowering maintenance costs can also be achieved by developing self‐regulating systems and innovative materials that reduce biofouling.^[^ ^29^, ^138^, ^139^, ^140^ ^]^ Cooperation between scholars, companies, and politicians is crucial to overcoming economic challenges. Material innovation, system integration, and stakeholder participation are key to making MFCs feasible in wastewater treatment and sustainable energy solutions.^[^ ^141^ ^]^

#### 4.1.2 Technical Challenges

MFCs face a variety of technical challenges when they are scaled up for widespread use. A key issue is that MFCs have a poor power density, which makes it difficult for them to compete with traditional energy sources.^[^ ^137^ ^]^ Furthermore, the economic viability of large‐scale MFC deployment is hampered by the high cost and short lifespan of electrode materials. Maintaining reliable and efficient biofilms is challenging, which becomes more challenging because of the complexity of the microbial communities inside MFCs.^[^ ^142^ ^]^ Moreover, electroactive bacteria's low growth rates may hinder the rapid formation of efficient biofilms. A major obstacle is still the optimization of electron flow processes between microorganisms and electrodes. Limitations in ion transport within the biofilm may result in poor performance and efficiency.^[^ ^143^ ^]^ Extreme conditions can negatively impact MFC functionality, hence controlling pH variations and salt levels is crucial. MFC reactors need to be carefully designed and scaled to ensure even substrate distribution and efficient mass transfer.^[^ ^7^ ^]^ The necessity for large surface areas to achieve feasible power outputs restricts the scalability of MFCs. Adaptable MFC designs are necessary to maintain reliable performance due to the diversity in wastewater composition. Predictive models for MFC performance in various circumstances are continuously being developed. It is still challenging to balance the trade‐offs between treatment efficiency and power generation.^[^ ^137^ ^]^ A thorough assessment of the use of resources and possible ecological impacts of large‐scale MFC deployment is necessary. Finally, in order to facilitate the integration of MFC technology into the current infrastructure, legislative frameworks and public acceptance must evolve.^[^ ^142^ ^]^ Various applications of MFCs with their corresponding technical challenges are shown in **Table** **3**.

**Table 3:** A summary of various MFC applications, including power generation and their corresponding challenges.

MFC configuration	Application area	Corresponding challenges	Key findings	Data on power generation	Refs.
DC‐MFC	Sensor	Low current output	This study presents a general method for integrating OECTs into MFCs and EFCs in order to enhance signal quality and intensify signals.	N/A	[158]
DC‐MFC	Power generation and wastewater treatment	Short‐term stability	In the best pH and temperature conditions, air‐cathode SCMFC electrodes worked better than traditional DCMFC ones, especially with Cu/Zn electrodes.	Mixed food cultures boosted power density in SCMFCs by ≈50%, reaching 3.31 W m^−^^2^ with composite anodes and 2.97 W m^−^^2^ with SS anodes.	[159]
DC‐MFC	Sustainable energy generation and wastewater treatment	Short‐term stability	During stable operation, the modified PPy–TiN/CS anode demonstrated a maximum O/P voltage of 653 mV, the highest PD of 2.44 W m^−2^, CD of 3.64 A m^−2^, and CE of 55.6%.	The modified PPy–TiN/CS anode demonstrated a maximum PD of 2.44 W m^−2^.	[160]
Terrestrial DC‐MFC	Power generation	Short‐term operational stability	The SMS electrode, made using the pasting and reinforcing method, generated 859 µW of power, outperforming the CF electrode, which produced 234 µW.	TMFC generated up to 859 µW of power.	[161]
Biomedia‐based DC‐MFC	Wastewater treatment	Small surface area and high resistance	The volume of catholyte production in MFCs with biomedia was 35% more than that in MFCs without biomedia.	N/A	[162]
DC‐MFC	Energy production and wastewater treatment	Short‐term stability	A PD of 219.3 mW m^−2^ and ≈70% TOC removal were achieved under reaction conditions.	A power density of 219.3 mW m^−2^ was achieved.	[163]
CW‐MFC	Pollution control and bioelectricity production	Issues due to different climates and wastewater types	The planted system reached a maximum voltage of 0.648 V and a power density of 232 mW m^−^^3^, while the unplanted system produced 0.537 V and 220 mW m^−^^3^.	A PD of 232 mW m^−3^ was produced by the planted system.	[164]
DC‐MFC	Bioelectricity production and wastewater treatment	Technical issues due to optimization of temperature and pH	A COD removal efficiency of 7.41% and a CE margin error of 18.65% were attained by the optimized MFC.	N/A	[165]
DC‐MFC	Carbon capture, nutrient recovery and removal, and bioelectricity production.	System instability	The system produced a 122.5 mW m^−^^2^ MPD, a 93.7% COD removal efficiency, and a 27.5% anode‐side TN removal efficiency at an optimal anodic pH of 6.	The maximum power density of the mMFC system was 122.5 mW m^−^^2^.	[166]
DC‐MFC	Electricity production	Low efficiency	For OPACC electrode‐based MFC, the study achieved the maximum PD of 163.84 ± 0.5 mW m^−2^ and CD of 372 ± 2.3 mA m^−2^.	The maximum PD of 163.84 ± 0.5 mW m^−2^ was attained by the MFC.	[167]
DC‐MFC	Blackwater treatment	COD conversion to electricity decreased.	With low methane production, the study produced MCD of up to 42 A m^−3^ and a 20% conversion of COD to electrical current.	N/A	[168]
SC‐MFC	Biomass production, sustainable pig slurry management, and renewable energy production.	Optimizing the concentration of pig slurry (PS) is necessary to minimize dilution and ensure water recirculation.	The method produced a voltage of 550 mV and a PD of 153 mW m^−2^, while also yielding notable decreases in COD and BOD.	The system attained power density of 153 mW m^−2^.	[169]
CW‐MFC	Bioenergy production, greenhouse gas reduction, and antibiotic disposal.	Scaling up issues	The CW‐MFC system yielded more chlorophyll, improved ammonium reduction, and removed more than 98% of antibiotics.	N/A	[170]
DC‐MFC	Wastewater treatment and bioelectricity production	Scaling up issues	A 1000 mL wastewater volume generated a significantly higher current of 83.41 µA (*p* < 0.05) compared to other studies, with COD and BOD residual values of 123.25 and 59.56 mg L^−1^, respectively.	The experimental results showed that the separator with 0.5 m NaCl produced a significantly higher current of 68.16 µA (*p* < 0.05).	[171]
DC‐MFC	Monitoring of natural attenuation in groundwater	Scaling up issues	The approach enables well‐informed decision‐making for efficiently monitoring and developing groundwater plume degradation techniques.	N/A	[172]
SC‐MFC	Wastewater treatment	Low power density	The maximal COD, TP, and phosphate degradation efficiencies achieved in the study were 76.3 ± 2.8%, 80.3 ± 3.3%, and 85.3 ± 3.5%, respectively.	The MFC's maximum power density was 1.9 ± 0.1 mW m^−2^.	[173]
SC‐MFC	Improvement of power production efficiency	Lower performance under varying operational conditions	A high open circuit voltage and power generation of 645 ± 24.5 mV and 424.51 ± 6.86 mW m^−2^ were demonstrated by the synthesized nanocomposite.	A high power output of 424.51 ± 6.86 mW m^−2^ was attained by the approach.	[174]

The adoption of MFCs on a broad scale requires the resolution of technical issues. Genetic engineering of microorganisms can be used to address the slow rate of electron transport from anode to cathode. The thickness of the biofilm is another way to enhance electron transport, as it may facilitate substrate movement and electron transfer. Increased internal resistance can be resolved without investing additional design costs by reducing the electrode distance between the anode and the cathode.^[^ ^35^ ^]^ Numerous investigations have documented the presence of metal/metal oxide nanocomposites embedded on the anode surface, which improve adherence of bacteria, electron transport, and ohmic loss reduction.^[^ ^52^, ^144^ ^]^ Optimizing electrode materials, area of surface, and catalysts is essential to resolving technical issues and boosting MFC performance, especially when it comes to reducing electrochemical losses and boosting cathodic reduction processes. Electro‐kinetic efficiency is improved by maintaining an ideal anode‐to‐cathode surface area ratio, which also helps to minimize ionic and species diffusion losses. With a 2 L single‐chambered MFC proving to be efficient for optimizing current production and promoting bacterial growth on biofilm‐covered anodes, scaling up can be accomplished by electrically stacking several MFC units or by expanding the anodic chamber volume.^[^ ^104^, ^145^ ^]^ MFC systems require technical advancements in order to function steadily across a broad temperature range. Regarding internal factors, parametric optimizations based on mathematical modeling and experimental design are beneficial. These factors include pH, cell biomass and substrate concentration, and proton mediator concentration. The development of efficient maintenance techniques for MFC devices is also essential.^[^ ^115^ ^]^

#### 4.1.3 Operational Challenges

MFCs are a promising technology for processing wastewater and producing sustainable energy, but a variety of operational issues prevent their widespread use. Since anodes must promote biofilm formation and enable effective electron transfer, which impacts MFC performance, choosing affordable electrode materials is a critical issue.^[^ ^29^ ^]^ The operational stress is increased by the requirement for continued optimization of operational parameters including substrate concentration and flow rate. System design complexity and integration issues are among the engineering and logistical barriers that come with scaling up MFCs.^[^ ^146^ ^]^ Reactor configuration optimization is complicated by factors like microbial variety and operating conditions, requiring careful design to find a compromise between scalability and performance.^[^ ^29^ ^]^ It is tough to ensure MFC performance stability over the long run; sustained efficiency over a long period requires extensive research and development. Microbial communities in MFCs are complex, which leads to performance variability and requires careful management to ensure reliable operation. Since material costs have a major impact on total system economics, developing affordable electrode materials is crucial to the economic feasibility of large‐scale MFCs. Multidisciplinary research integrating materials science, engineering, and microbiology is needed to address these issues and create integrated solutions.^[^ ^147^ ^]^ Pilot‐scale studies that provide insights into scalability and performance in the real world are essential for bridging the gap between laboratory research and commercial application. The viability of large‐scale MFC implementations must also be evaluated through economic feasibility studies, which ensure that the technology is both environmentally and financially sustainable.^[^ ^29^ ^]^ Microbial activity depends on pH, resulting in MFCs' susceptibility to variations in pH may result in instability in their function. Low power density is still a major barrier to MFCs' broad use, which restricts their potential for energy production. Low current output may be the consequence of the slow kinetics of electron transport between microorganisms and electrodes. It is necessary to address reactor design and size scale‐up issues in order to increase efficiency and integrate with current infrastructure.^[^ ^148^ ^]^ Table 3 summarizes the operational challenges of various MFC applications.

Several approaches have been presented to overcome the operational challenges faced by MFCs. The low power output can be addressed by improving electron transfer efficiency by electrode design and material optimization. Maintaining pH stability through the use of effective buffering systems can improve performance and output power.^[^ ^149^, ^150^ ^]^ In order to enhance the performance and output power of MFCs, membrane‐less designs have been developed to remove the resistance connected to proton exchange membranes.^[^ ^151^ ^]^ Moreover, numerous units and modular designs have been proposed as ways to boost power outputs. Integrating MFCs into wastewater treatment systems solves the issue of substrate availability by treating waste and provides a substrate for the production of energy.^[^ ^29^, ^152^, ^153^ ^]^ The efficiency issue caused by the oxygen crossover from the cathode to the anode can be reduced by optimizing reactor design and using gas diffusion barriers.^[^ ^151^, ^154^ ^]^ Optimizations of the operational parameters are crucial to achieving the highest feasible energy recovery, substrate degradation, and CE because these factors are directly related to the circuitry and MFC designs. Modifying the substrate's properties, the inoculation techniques, pH, hydraulic retention time, operation mode, and temperature are some of these parameters.^[^ ^155^, ^156^, ^157^ ^]^ Finally, multidisciplinary collaboration is necessary to integrate MFCs with current infrastructure in a way that ensures smooth implementation and operation.^[^ ^29^ ^]^

### 4.2 Important Factors Affecting the Efficiency of MFCs

#### 4.2.1 Electrode Material

The electrode, one of the major elements in the design of an MFC, is what primarily determines the performance and cost of an MFC. Thus, designing electrodes provides the greatest challenge to the scalability and cost‐effectiveness of MFC technology.^[^ ^52^ ^]^ Electrode materials can enhance an MFC's performance and function, due to the fact that different anode materials produce variable activation polarization losses. The use of inappropriate anode materials can have a detrimental effect on MFC performance. Low conductivity in anode materials can reduce electrochemical efficiency by increasing resistance and impeding electron transfer. Bacterial adhering is restricted by a small surface area, which also reduces the amount of bacterial active sites and blocks electron flow. Gold, silver, and copper are demonstrations of non‐biocompatible materials that can corrode and limit the growth of bacteria. The physical stability of the electrode may be affected by low mechanical strength, thermal instability, and swelling from corrosion. Additionally, the overall expense of MFCs is increased by the use of costly components like gold and silver.^[^ ^20^, ^145^, ^175^, ^176^ ^]^ For the manufacture of anodes and cathodes, Pt black and Pt electrodes are superior to graphite, carbon‐cloth, and graphite‐felt electrodes, but they are extremely costly.^[^ ^57^ ^]^ For large‐scale applications, it is not economically feasible to utilize an electrode material that is very efficient, like platinum. As a result, MFC‐related research currently places a greater priority on investments that are more cost‐effective.^[^ ^26^ ^]^ For higher power production, it is greatly beneficial to modify the anode's electrode with a catalyst or nanomaterial that can assist the growth of biofilms and increase electron transfer rates. As an example, employing G. sulfurreducens as an inoculum, magnetite nanoparticles improved the output current in the MFC. The study revealed that the nanoparticles improved the conductivity of the biofilm–electrode contact, hence enhancing the electron transfer process.^[^ ^62^ ^]^ Schroder et al.^[^ ^57^ ^]^ discovered that a platinumized carbon‐cloth anode could achieve a current of 2–4 mA. In contrast, the unmodified carbon‐cloth did not exhibit any microbially facilitated current flow under the same operating conditions, using a standard glucose medium at 0.55 mmol l^−1^ in a vibration culture of *E. coli*.

The development of various materials with increased power density and efficiency was facilitated by advancements in electrode material design. A variety of electrodes have been made from carbon‐based materials because of their high porosity, large surface area, and superior electrical conductivity. In recent years, several nanocomposites with improved characteristics have been proposed and produced to increase electrode performance.^[^ ^20^, ^177^, ^178^ ^]^ Stainless steel is a very efficient material for increasing MFCs' power density and coulomb efficiency. By introducing stainless steel to different electrode materials, noticeable improvements were thereby achieved. Graphene has also been employed widely as an electrode material in MFCs due to its excellent properties of high conductivity and large surface area. In MFCs, graphene‐modified stainless steel mesh anodes showed a power density of 2668 mW m^−2^. Additionally, a modified electrode based on multi‐walled carbon nanotubes was produced to enhance MFC performance.^[^ ^20^, ^179^ ^]^ Natural biomass‐based electrode materials provide an economical and environmentally friendly way to improve MFC performance. High‐temperature carbonization of plant waste produces highly conductive porous structures that promote electron transfer and bacterial colonization.^[^ ^59^, ^180^ ^]^ Metal oxide/carbon composites improve electron transfer efficiency by addressing the limitations of individual materials, such as limited carbon conductivity and metal corrosion. Although pure metals such as gold, silver, titanium, and platinum have good conductivity, their weak bacterial adherence and high cost prevent widespread application. Metal oxides, such as ZnO, Ag, and TiO₂, offer a less expensive substitute with similar catalytic performance and less bacterial cell toxicity. In contrast to traditional graphite anodes, TiO₂‐modified loofah sponge carbon anodes for instance showed a 200% improvement in energy generation. Metal oxides can be used with conductive polymers or carbon‐based materials to improve electron transport and increase MFC efficiency.^[^ ^56^ ^]^

#### 4.2.2 Proton Exchange Membrane System

The MFC output power is influenced by the proton exchange system's impact on the concentration of polarization loss and the internal resistance of the MFC system. The anolyte diffuses across the membrane to the cathode, fouling the membrane and inhibiting proton flow to the cathode, reducing the MFC's power output. Moreover, the ability of the bacteria to produce electricity in MFC is impacted by the entrance of the catholyte into the anode chamber. Over a comparatively broad range, an increase in PEM surface area causes a decrease in MFC internal resistance. The most prevalent Nafion has a very selective protons permeability. So far, even with Nafion, the side effects of other cations are crucial for MFC operation.^[^ ^26^, ^57^ ^]^ Nafion refers to the cation exchange membrane which is most commonly used for proton exchange due to its distinctively high proton and conductivity. The negative charges on Nafion facilitate the transfer of undesired cations, reducing the pH of the anode compartment and disrupting the microbiological potential at the anode. Instead, an increase in the cathode's pH causes a decrease in cathode efficiency, which has an impact on the MFC's working efficiency. The degradation of the PEM can occur due to factors such as improper fabrication, operational conditions, and material properties, leading to reduced proton transport and compromised MFC efficiency. Additionally, degradation can occur through mechanical, thermal, or chemical/electrochemical mechanisms. Nafion's tendency to lower the pH at the anode and raise it at the cathode, along with its reduced proton conductivity under high temperatures and low humidity, contributes to decreased microbial potential and reduced overall efficiency.^[^ ^57^, ^181^, ^182^ ^]^ On the other hand, anion exchange membranes boost the MFC's power output and efficiency by improving the transfer of protons while limiting the transfer of other cations, maintaining the pH gradient.^[^ ^183^ ^]^ In an MFC implanted with G. metallireducens, Min et al.^[^ ^184^ ^]^ investigated the PEM performance and salt bridge. The output power was 2.2 mW m^−2^ utilizing the salt bridge, which is less than the result using Nafion. According to research findings, MFCs having PEMs with large surface areas produce less internal resistance resulting in higher power production. Some studies show that MFCs without a PEM have a higher power output than MFCs with a membrane for a certain amount of time. One of the PEM's drawbacks is also its higher cost. Hence, we may imagine membrane‐less MFCs being used in large‐scale applications in the future.^[^ ^62^ ^]^

Recent advances in MFC membranes focus on developing affordable alternatives to PEMs based on Nafion. Earth‐abundant materials and ceramic‐based membranes have been explored; in ceramic membranes, proton conduction is improved by the introduction of montmorillonite, a mineral with a high cation exchange capacity. Furthermore, graphene oxide has been used in PEMs because of its capacity to serve as a scaffold for sulfonyl groups and to offer a porous structure that promotes efficient proton transfer. In order to improve MFC performance, these developments emphasize the necessity of proton exchangers that maintain porosity and support the hopping mechanism.^[^ ^185^, ^186^, ^187^ ^]^ Additionally, an innovative, economical, and environmentally friendly strategy for high proton‐conducting membranes in MFCs has been established by combining clay and activated carbon from coconut shells (ACCS). A possible substitute to conventional Nafion‐based PEMs, this composite membrane has high porosity and excellent specific surface area. The feasibility of PEM has been evaluated through systematic characterization studies that include IEC, swelling ratio, acetate diffusion, oxygen diffusion, proton conductivity, and intake of water. The effectiveness of the ACCS/clay‐based membrane in producing electricity and treating wastewater was evaluated in MFCs and compared with the industry conventional Nafion 117 membrane, demonstrating its potential as a feasible, affordable alternative.^[^ ^65^, ^188^, ^189^, ^190^ ^]^

#### 4.2.3 Electron Transfer Mechanism

Electron transfer occurs as a result of proteins and other biomolecules found in bacteria mediating the donation and electron acceptance between the electrodes and bacteria. Extracellular electron transfer is primarily mediated by two different mechanisms: mediator‐less or DET and indirect or MET. Microorganisms do not have any electrochemically active proteins for the transfer of electrons to the anode on their surface in METMFC. Mediator‐less MFCs operate without external electron carriers.^[^ ^183^ ^]^ The electrode surface and the bacterial cell's membrane‐bound cytochrome must have direct physical contact in order for the DET mechanism to function. Electron transfer proteins assist in the transfer of electrons from the cytoplasmic to the bacterial cell's outer membrane.^[^ ^15^, ^191^ ^]^ The electron transfer mechanism in MFCs directly impacts their performance by affecting how efficiently electrons are transferred from bacteria to the electrode. In DET, bacteria transfer electrons through outer membrane cytochromes, conductive pili, and nanowires, requiring close contact between the microbial membrane and the anode. Species like Geobacter and Shewanella enhance current densities due to their extensive networks of cytochromes. However, only bacteria in direct contact with the anode can participate in DET, limiting the process. Conductive biofilms can help overcome this limitation by allowing electron transfer through thicker layers. Some bacteria rely on MET, using redox carriers to shuttle electrons to the anode. Mediators, whether endogenous or exogenous, must meet certain criteria, such as non‐toxicity and good solubility, to efficiently facilitate electron transfer and improve MFC performance.^[^ ^192^, ^193^, ^194^, ^195^ ^]^ The soluble molecules used in the METmechanism fall into two categories: exogenous redox mediators and soluble electron carriers produced by bacteria.^[^ ^52^ ^]^ Microbes utilize wastewater to produce an electric current in order to fulfill the energy demand in an eco‐friendly manner. This is accomplished through these electron transfers. Exogenous mediators have been used much less commonly over the years because of their instability and toxicity, yet this method is still reasonably popular to enhance MFC efficiency. As a result, effective electron transfer is crucial for the proper operation of MFCs.^[^ ^15^, ^183^ ^]^

#### 4.2.4 Substrates

The composition of the substrate and its susceptibility to microbial action plays a significant role in energy production in MFCs. Currently, a variety of organic products, from the simplest to the most complex, are used as substrates for the advancement of MFCs.^[^ ^196^ ^]^ One of the elements that affect the efficiency of the electrochemically active microorganism population in the anodic chamber is the substrate. When mixed culture is utilized as the inoculum, it also identifies the existence of the dominant bacterial population. It is common practice to maintain homogeneity using pure substrates like acetate, glucose, and butyrate. Simple organic compounds like glucose and acetate enhance electron transfer and power output, while complex substrates such as wastewater require additional enzymatic breakdown, reducing efficiency. Substrate salinity also impacts performance, with optimal salinity improving power density but excessive salinity negatively affecting MFC function. The concentration of the substrate must be balanced, as too much can lead to microbial inhibition, pH imbalance, or toxicity. Additionally, different substrates shape microbial community composition and biofilm formation, affecting system stability and electrode interactions.^[^ ^144^, ^197^, ^198^, ^199^ ^]^ In the anode compartment, exoelectrogenic bacteria use nitrogen‐rich substrates, such as cysteine and proteins, to produce electricity.^[^ ^200^ ^]^ Other complex substrates, including starch, chitin, cellulose, molasses, dextran, and pectin, have also been employed due to the expanding interest in MFCs. Moreover, wastewater containing organic compounds from various industrial sources has been employed as a substrate for the production of bioelectricity.^[^ ^196^ ^]^ However, recent research, indicates that glucose and acetate are the substrates that are used the most commonly, with acetate producing the highest results. This prompted the need to examine these two substrates for the generation of power and the treatment of wastewater.^[^ ^26^ ^]^

#### 4.2.5 Effect of pH

The pH is another essential factor for MFC, due to the participation of numerous microbial populations that are sensitive to pH fluctuation. As the pH approaches 7, the MFC microbial communities can grow new active biofilm and survive in challenging conditions. The performance of MFC in both continuous and batch modes of MFC function is controlled by the pH of the substrate.^[^ ^201^ ^]^ The pH and the performance of MFC operation are influenced by the rate of proton transfer to the cathode from the anode. Proton formation at the anode, which causes anode acidification, is the result of the proton's slow transport rate. Raising the pH at the cathode results from the usage of proton in oxygen reduction and a slower rate of proton transfer. The loss in oxygen reduction caused by this increase in pH at the cathode causes a decrease in power production. Moreover, maintaining pH within an appropriate range is crucial for controlling microbial metabolism and has a direct impact on the MFC's performance to generate power.^[^ ^52^, ^183^ ^]^ Since the pH of the environment has a direct impact on the metabolism activity of microorganisms, the continuous shift would cause both the cathode potential and the voltage output to fluctuate. This issue might be resolved if non‐membrane bio‐cathode MFCs were used, as oxygen could diffuse directly into the anode.^[^ ^202^, ^203^ ^]^ Several studies indicate that a pH of 7 is more favorable to microorganisms and that a lower pH has a detrimental impact on the formation of biofilms. Similarly, a pH level of 7 is favorable for more effective wastewater treatment and higher power generation.^[^ ^201^ ^]^

#### 4.2.6 Effect of Temperature

One of the key elements influencing MFC's ability to produce electricity is temperature. A wide range of temperatures can be effectively operated by MFCs. Power density, chemical oxygen demand removal, electrode potential, Columbia efficiency, and internal resistance of MFCs are all impacted by operating temperature. The temperature has an exponential effect on microbial activity, which has an impact on the MFC's output of power. It is well known that raising the temperature maximizes microbial activity.^[^ ^50^ ^]^ The temperature must be raised in order to improve the power output of MFC, which will lead to more bacterial activity. The performance of MFCs in removing COD and producing power is found to be directly influenced by temperature. In contrast to ohmic resistances, power density increases as temperature rises. It is also demonstrated that there is no relationship between membrane permeability and power output or temperature increase. However, there is a linear trend showing that higher temperatures cause less ohmic resistance. This might be the result of the ionic conductivity that temperature rises cause.^[^ ^204^, ^205^ ^]^ As a result, some literature has demonstrated that temperature plays a significant role in the initial biofilm production at the MFC's startup. Moreover, the MFC starts up faster at higher temperatures, which often promotes the production of stable biofilms.^[^ ^26^ ^]^ Kinetics, activation energy, solution conductivity, electrode potentials, and microbial behavior are all impacted by temperature. Every microorganism has a preferred temperature for its development and metabolic processes. Biofilm formation and bio‐electrocatalytic activity can both be improved at temperatures between 30 and 45 °C. While a slight temperature drop throughout the MCF process can reduce heating costs, it has minimal to no impact on the production of electricity.^[^ ^35^ ^]^ There are multiple optimum temperatures reported in different studies because different microorganisms have considerably different temperatures. Hence, the temperature needs to be adjusted for a specific type of effective wastewater treatment and energy production by the MFC.^[^ ^201^ ^]^ The highest efficiency of MFC was found to be realized at 35 °C, and it was noticed that an increase in power density was achieved as the temperature increased from 24 to 35 °C.^[^ ^183^ ^]^

#### 4.2.7 Shear Stress and Feed Rate

Hydraulic retention time, flow rate, and shear stress are important elements that must be taken into consideration, before MFCs may be employed successfully in wastewater treatment plants. The feed rate controls the availability of nutrients and electron donors for microbial metabolism. A higher feed rate ensures a steady supply of substrates, boosting power generation, but an excessively high rate can lead to inefficient substrate utilization and washout of active microbes. Conversely, a low feed rate may limit microbial growth and slow down electron production.^[^ ^57^, ^206^ ^]^ The increased flow rate causes the increased power output. Power density decreases when a very high flow rate is used. This demonstrates that the best performance can be achieved when the microbial population has the time to develop when nutrient usage is optimal, and when substrate hydrolysis is at its maximum.^[^ ^57^ ^]^ The optimum flow rates are recognized to result in well power densities, but even there is a negative effect in higher flow rates. Therefore, it is believed that optimum can only be achieved if the microbial population has proliferated and conditions for nutrient capture and substrate hydrolysis are most favorable. Shear stress, generated by fluid flow, influences the stability and thickness of the biofilm on the electrode surface. Moderate shear stress can enhance mass transfer and prevent biofilm detachment, improving electron transfer efficiency. However, excessive shear stress may damage the biofilm, reducing microbial activity and power output.^[^ ^57^, ^206^ ^]^ Greater shear rates that are less than tensile strength result in thicker, electrons that can be transmitted more quickly by denser biofilms, raising the power density.^[^ ^207^ ^]^ Researchers recently proposed a technique to revitalize biofilms by using shear stress of 9.34 mPa to rejoin dead cells from a biofilm with a decrease in polarization resistance. Thus, the operating conditions of MFCs affect their MFC performance. These operating parameters should be optimized for optimal performance.^[^ ^15^ ^]^

#### 4.2.8 External Resistance

The external resistance (RE) is a crucial tool for power dissipation by regulating anode and output power, which are controlled by the electron flow through the external circuit of the MFC. The RE regulates the interaction between the cell voltage and current, which is governed by the distinctions between the cathode and the anode potential. When the external resistance is too high, the current flow decreases, leading to lower power generation despite a higher voltage. Conversely, if the external resistance is too low, excessive current flow can cause internal losses, reducing the overall voltage and efficiency. Examining the ecosystem of exoelectrogenic and bioelectrochemistry aspects of MFC could better facilitate ER. Moreover, it affects the microbial populations' composition within the biofilm. The adaptability of the microbial population in the anodic chamber is another factor that affects MFC performance at a specific external resistance.^[^ ^91^, ^152^, ^201^, ^208^ ^]^ The efficiency of substrate removal is increased because the lesser resistance makes it easier for electron flow to the cathode from the anode, facilitating the respiration of microbial electrons on the anode. Conversely, the larger resistance increases the power harvest by reducing electron transport toward the cathode while retaining a high potential difference. Previous research indicated that the maximum power generation occurred when the internal and external resistances were equal. Proper resistance selection helps balance electron transfer, ensuring efficient microbial activity and energy conversion. The optimization of internal resistance can increase the performance of MFC.^[^ ^201^, ^209^ ^]^

### 4.3 Commercialization

MFC technology's robustness, lack of reproducibility of outcomes, and system stability issues make commercialization still challenging. The risk associated with the commercialization of MFCs has not yet been thoroughly established in full‐scale operational applications. For commercialization, there are a number of other factors to take into account, such as the stability and reliability of system performance. In the recovery market and wastewater treatment, major upfront business is generally limited.^[^ ^134^ ^]^ The laboratory‐scale research on MFC has received a lot of concerns recently, however, there is not enough coverage of commercial‐scale applications. MFC technology has seen significant advancements over recent years. However, MFC commercialization is still hampered by numerous restrictions. Scaling up MFC is one of the principal challenges.^[^ ^52^ ^]^ The key challenges to the commercialization of MFCs are the lack and higher cost of Pt.^[^ ^210^ ^]^ The cost of their small‐scale manufacture and components are the major challenges to an MFC's development into commercialization. Large‐scale commercialization is also another issue of MFCs in the business industry. Further investigation into material production stability is required to address these issues. These materials utilized in MFC design are susceptible to electrolyte corrosion. The quality and cost of the materials are crucial issues that need to be focused on research before the MFC technology can be commercialized.^[^ ^211^ ^]^

### 4.4 System Complexity

MFC is a multifunctional technology that depends on contributions from electrochemistry, bioengineering, chemical engineering, material science, and other applied engineering disciplines to be an effective wastewater treatment system. Operating factors such as heavy metal concentration, pH, rate of organic loading, etc., and contamination presented to the bacterial fermentation due to the presence of some of the constituents may result in poor performance of MFC while treating real industrial wastewater. Hence, operating parameters in MFCs at the laboratory scale must be optimized before scaling‐up experiments. MFC is subjected to unpredictably changing conditions in the real wastewater environment even in field applications, which is anticipated to drastically change its performance.^[^ ^134^ ^]^ The system is complex and faces challenges during scaling‐up due to the various processes, including biological systems, electrocatalytic issues, financial barriers, microbial constraints, and self‐sustainability. Future research must address microbial contamination challenges to enhance the electron transfer rate and overcome electrochemical constraints. Additionally, researchers need to tackle cost issues related to membrane and electrode materials, system complexity, and operational limitations in lab‐scale reactor designs.^[^ ^104^, ^212^ ^]^

## 5 Future Prospect of Microbial Fuel Cells

From a future perspective, MFCs are viable and environmentally friendly substitutes for resolving some global issues associated with the energy crisis and environmental degradation. However, a significant barrier to the development of MFCs as renewable energy sources has been the low and unstable power output. As a result, a recent focus has been on the lack of adequate liability for the production of bioelectricity.^[^ ^104^ ^]^ By the optimization of several operational parameters and design aspects, MFC technology has achieved significant advances over the last decade in terms of enhanced efficiency as well as output power, and this has encouraged strong global research in this field. In addition to producing bioelectricity, supported developments and ongoing improvement have established different practical uses for MFC technology in several fields.^[^ ^51^, ^99^ ^]^ Scaling up MFC systems is required to make them suitable to be utilized in real‐world applications. To accomplish this, it is essential to expand the MFC reactor's size and capability for treatment to a practical level. Many challenges need to be overcome before this technology can be commercialized. Several potential applications outside of energy production have been developed because of the ongoing research and development on MFC technology.^[^ ^31^ ^]^ Before MFCs can be scaled up, their performance should be increased as their deployment is still unfeasible. Therefore, the researchers' overall goal is to develop and evaluate biocompatible electrodes and membranes, innovative configurations, and low‐cost catalysts for increasing electron acceptor reduction.^[^ ^213^ ^]^ The performance of MFCs can be enhanced through optimization of the temperature, substrates, and pH in the chambers, as well as by modifying electrode resistance, electrode materials, the electron transfer mechanism, and separation membranes. The mechanisms of the electron pathway to be comprehended and the loss of electron transfer should be minimized in order to optimize the potential of MFC technologies, in future research. The formation of biofilms and electron coupling may be enhanced by using the appropriate materials for the electrodes.^[^ ^15^, ^155^, ^209^ ^]^ It is essential to modify the anode and cathode materials because the electrode material plays a vital role in the performance development of MFCs. The manufacturing cost should be minimized for MFC development. An effective approach for enhancing power production has been to modify the electrode using various materials.^[^ ^213^, ^214^ ^]^ Several chemical modifications to the membrane are employed to greatly improve the electron transfer rate and MFC power density. The electrodes and various plasma membranes have been modified to enhance the performance of MFCs using carbon nanotubes, metal nanoparticles, conductive polymers, and other metal‐based nanostructures.^[^ ^215^ ^]^ The primary opportunity for MFC utilization is the fact that the output power of MFC systems has significantly increased since its early reports in 2004. MFCs have been demonstrated in numerous studies to be an effective candidate for various contaminant removal, the production of bioelectricity and biohydrogen, the treatment of wastewater, the development of biosensors, and robotics.^[^ ^216^ ^]^ MFC technology can be coupled with traditional systems to increase the removal of contaminants and the recovery of resources from wastewater.^[^ ^104^ ^]^ MFCs seem to have a promising future due to the abundance of applications that can be used with them. Despite the poor electrical output that certain MFC designs produce, literature has demonstrated that there is no limit to the diversity of MFCs. Each modification draws these devices closer to their application in everyday life and proposes a novel strategy for increasing their efficiency.^[^ ^215^ ^]^

MFC integration with renewable technologies provides an effective way to produce environmentally friendly energy and enhances grid stability. The potential for MFCs to be used in wastewater treatment is one of the most interesting. By breaking down organic waste and producing power at the same time, they can lower pollution and energy expenses. Hybrid systems, such as solar‐MFC and wind‐MFC configurations may ensure steady power production by using MFCs during off‐peak hours and renewable resources during peak availability. MFCs can also help produce hydrogen, providing an alternative to clean fuel alternatives. They have the potential to power small‐scale grids, smart city infrastructure, remote sensors, and off‐grid energy solutions. MFCs could help with bioremediation and carbon capture, which would solve environmental and energy‐related issues. However, advancements in nanomaterials are required to overcome barriers including low power output, scalability, and cost efficiency. MFCs may play a significant role in the transition to cleaner, more sustainable energy if these issues are addressed.^[^ ^12^, ^14^, ^198^, ^217^, ^218^ ^]^

Nanomaterial advancements have greatly improved MFC possibilities by enhancing power output, improving overall system stability, and increasing electron transfer efficiency. With their high conductivity, large surface area, and remarkable biocompatibility, nanomaterials such as metal nanoparticles, graphene, carbon nanotubes, and nanocomposites improve bacterial adhesion and electron transfer at the anode. Furthermore, the MFC efficiency can be improved by nanostructured cathode catalysts, such as metal oxide and platinum nanoparticles, which enhance oxygen reduction processes. Nanomaterial integration in MFCs also makes it possible to develop scalable, adaptable, and compact bioenergy systems that can be used for remote power, biosensors, and wastewater treatment. As research advances, the integration of nanotechnology and MFCs has great potential for environmentally friendly energy production and remediation.^[^ ^52^, ^219^, ^220^ ^]^

## 6 Conclusion

MFCs are the best possible option and a crucial field for innovation and research for sustainable future development and growth in the modern global economy. The diverse possible applications of MFCs are emerging all over the world to resolve environmental pollutants and meet the energy demand, which is discussed in this review paper. This paper presents the potential applications such as biohydrogen production, wastewater treatment, bioelectricity generation, removal of hazardous wastes, biosensors, robotics, and other applications (e.g., dye decolorization, bioremediation, carbon capture, sanitation, portable electronic appliances, etc.). This review article also focuses on some of MFC's challenges like large‐scale applications, economics, commercialization, system complexity, and several operational factors. Due to the poor power generation and high operating costs, large‐scale MFC practical applications are currently hampered. To increase the power output on large‐scale applications, suitable nanomaterials should be utilized in membrane and electrode fabrication at a low cost. The power output of MFCs to be utilized in real‐world applications can also be improved by expanding the reactor size of MFCs. Before the MFC technology can be commercialized, research must be focused on addressing the quality and cost of the materials in order to minimize system complexity and economic concerns. Moreover, important operational factors including proton exchange system, pH, temperature, electron transfer mechanism, substrate, and external resistance should be optimized to enhance the efficiency of MFCs. Future prospects of MFCs are also highlighted in this paper. Finally, by optimizing the operational parameters of MFCs and utilizing suitable materials in electrode fabrication, a cost‐effective and high‐performance MFC technology is possible in the modern world to resolve the current energy shortage and environmental contamination.

## Conflict of Interest

The authors declare no conflict of interest.
